# Supplementary material for: Legacy of draught cattle breeds of South India: Insights into population structure, genetic admixture and maternal origin
Source: PLoS One. 2021 May 24;16(5):e0246497. doi: 10.1371/journal.pone.0246497 (PMC8143428; doi:10.1371/journal.pone.0246497)

S1 Fig. The test for selective neutrality at 27 microsatellite marker loci. Selection detection based on the *F*_ST_ outlier approach using LOSITAN showed that two marker loci (HEL13 and HEL5) deviated from selectively neutrality


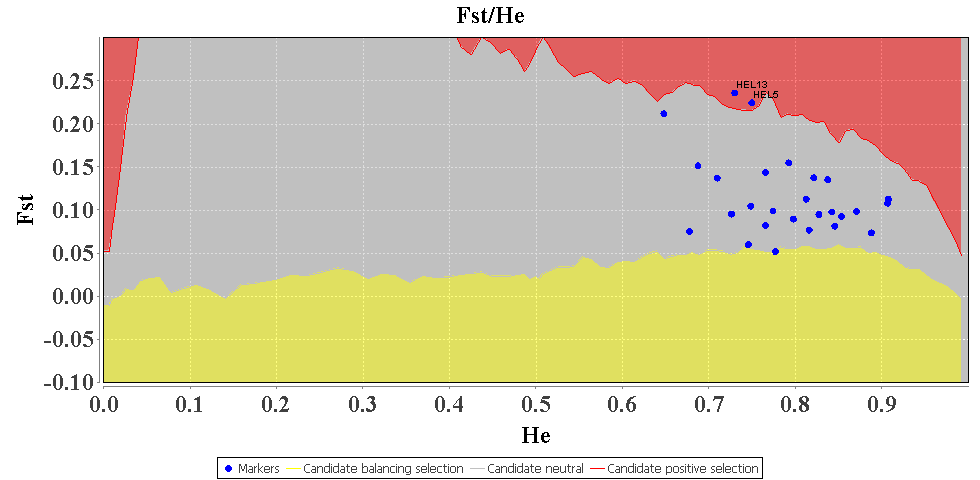

Supplement: S1 Fig — Selection detection based on the FST outlier approach using LOSITAN showed that two marker loci (HEL13 and HEL5) deviated from selectively neutrality. (DOCX) [file pone.0246497.s001.docx]
