# Supplementary material for: Legacy of draught cattle breeds of South India: Insights into population structure, genetic admixture and maternal origin
Source: PLoS One. 2021 May 24;16(5):e0246497. doi: 10.1371/journal.pone.0246497 (PMC8143428; doi:10.1371/journal.pone.0246497)

S2 Fig. Determination of correct number of clusters in Bayesian STRUCTURE analysis (Evanno et al. 2005) (a) Mean L (K) over 10 runs for each K value of 1 to 15 (b) Distribution of ∆K with the modal value (K=2) indicating the true K or the uppermost level of structure


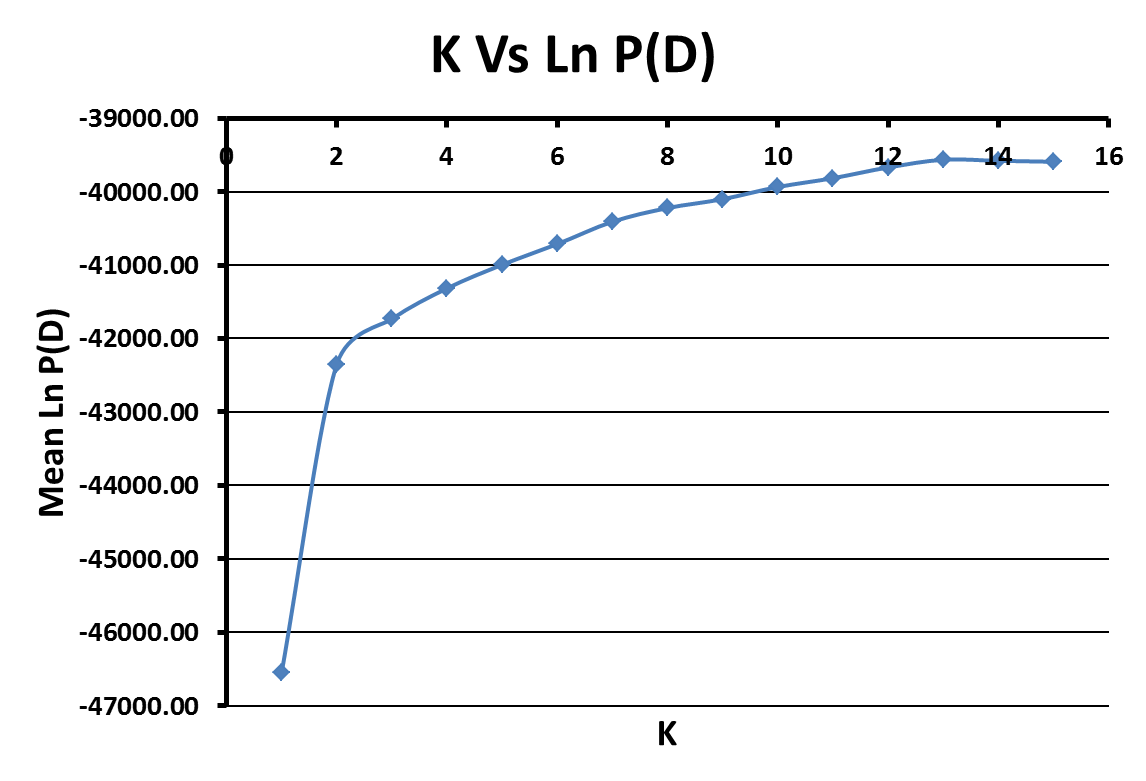


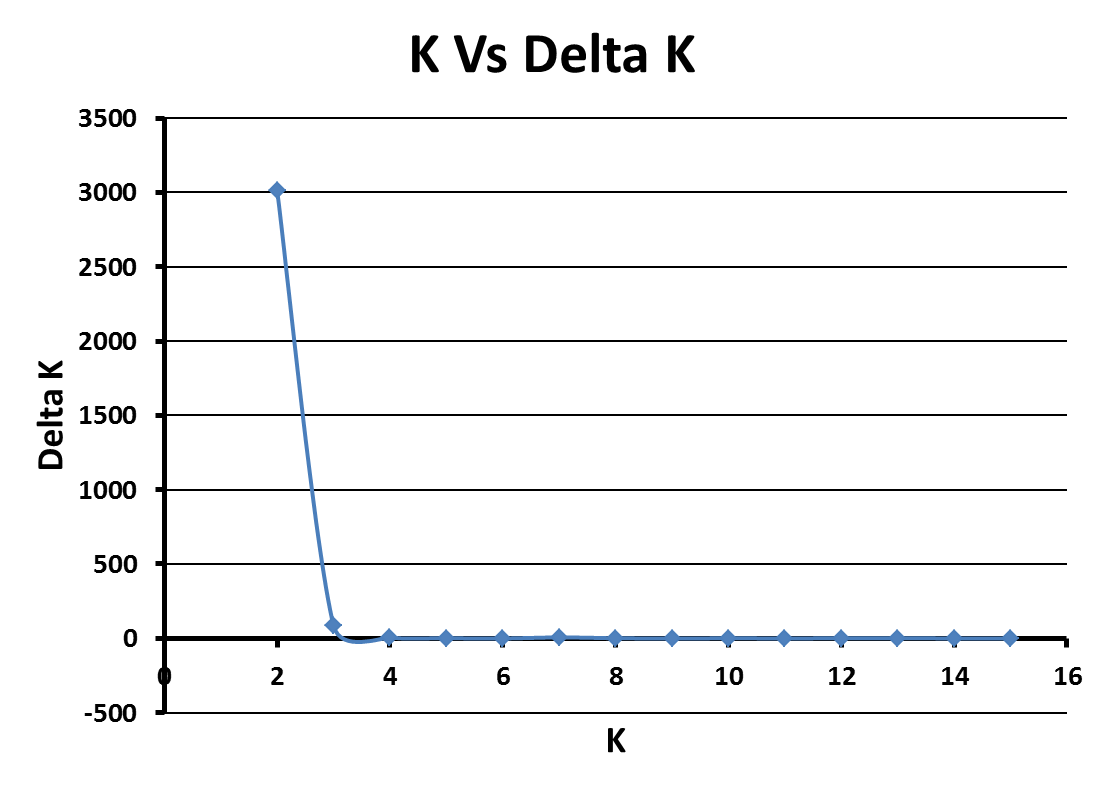

Supplement: S2 Fig — Determination of correct number of clusters in Bayesian STRUCTURE analysis (Evanno et al. 2005) (a) Mean L (K) over 10 runs for each K value of 1 to 15 (b) Distribution of ΔK with the modal value (K = 2) indicating the true K or the uppermost level of structure. (DOCX) [file pone.0246497.s002.docx]
