# Supplementary material for: Legacy of draught cattle breeds of South India: Insights into population structure, genetic admixture and maternal origin
Source: PLoS One. 2021 May 24;16(5):e0246497. doi: 10.1371/journal.pone.0246497 (PMC8143428; doi:10.1371/journal.pone.0246497)

S3 Fig. Maximum likelihood tree of mitochondrial DNA haplotypes of draught type zebu, taurine and crossbred cattle from South India


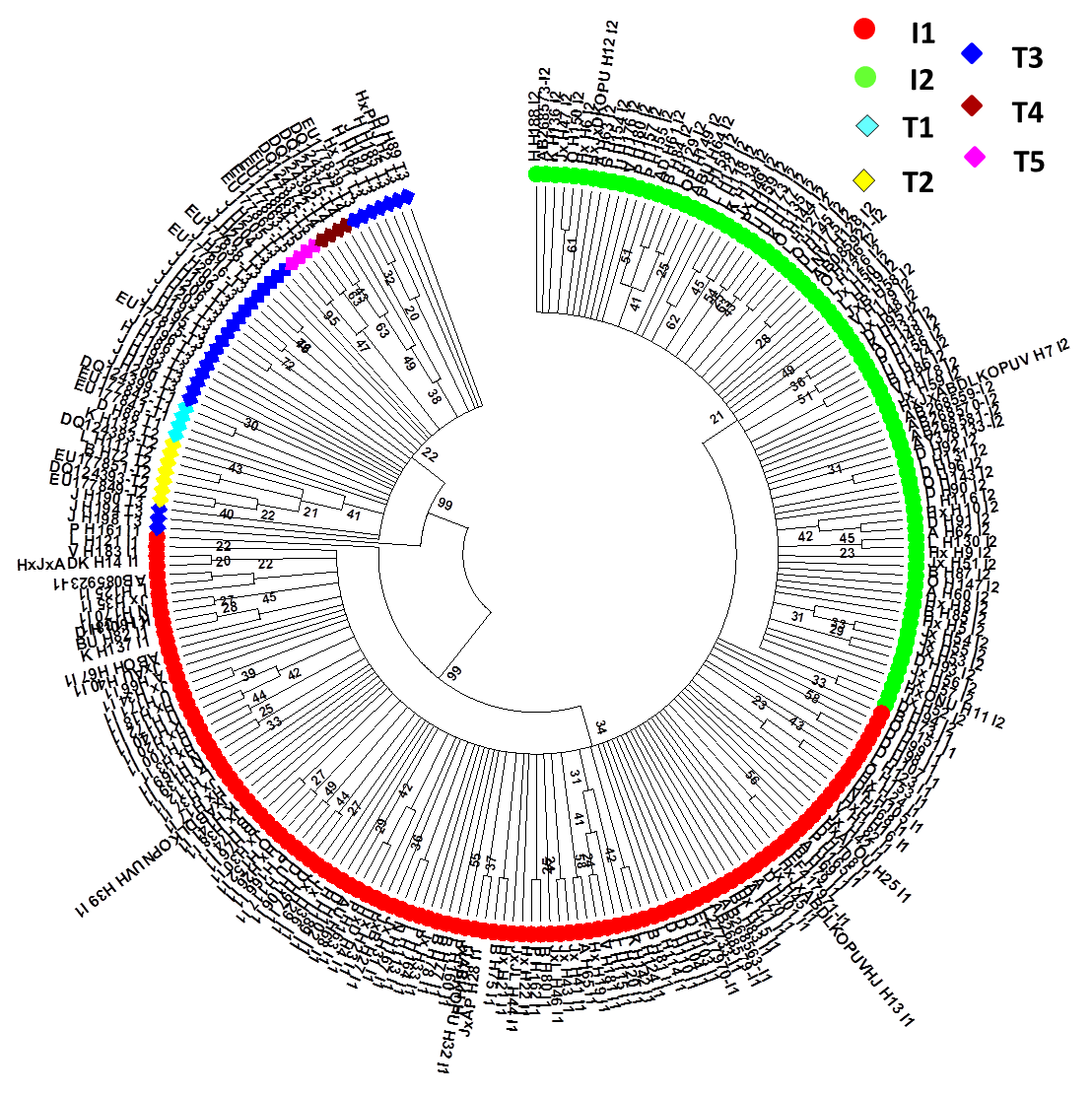

Supplement: S3 Fig — (DOCX) [file pone.0246497.s003.docx]
