# Supplementary material for: Legacy of draught cattle breeds of South India: Insights into population structure, genetic admixture and maternal origin
Source: PLoS One. 2021 May 24;16(5):e0246497. doi: 10.1371/journal.pone.0246497 (PMC8143428; doi:10.1371/journal.pone.0246497)
Supplement: S1 Table — (DOCX) [file pone.0246497.s004.docx]

S1 Table. Details of 27 FAO recommended microsatellite loci used to evaluate South Indian draught cattle breeds

| S.No | Locus | Multiplex Panel | Annealing Temperature | Dye | Allele Size Range |
| --- | --- | --- | --- | --- | --- |
| 1 | CSRM60 | 1 | 60°C | FAM | 87-109 |
| 2 | CSSM66 | 1 | 60°C | FAM | 177-197 |
| 3 | HEL1 | 1 | 56°C | HEX | 96-114 |
| 4 | INRA63 | 1 | 56°C | HEX | 173-183 |
| 5 | BM1824 | 2 | 61°C | ATTO550 | 183-197 |
| 6 | ETH152 | 2 | 60°C | FAM | 189-199 |
| 7 | HAUT27 | 2 | 54°C | HEX | 140-150 |
| 8 | INRA05 | 2 | 54°C | FAM | 134-148 |
| 9 | BM1818 | 3 | 60°C | HEX | 256-272 |
| 10 | ETH3 | 3 | 63°C | FAM | 99-125 |
| 11 | HEL9 | 3 | 56°C | ATTO550 | 155-175 |
| 12 | ILSTS006 | 3 | 54°C | FAM | 284-300 |
| 13 | TGLA53 | 3 | 55°C | HEX | 153-187 |
| 14 | HAUT24 | 4 | 53°C | HEX | 103-125 |
| 15 | HEL5 | 4 | 54°C | FAM | 148-164 |
| 16 | INRA032 | 4 | 56°C | ATTO550 | 164-208 |
| 17 | SPS115 | 4 | 61°C | FAM | 243-255 |
| 18 | ETH185 | 5 | 65°C | ATTO550 | 224-252 |
| 19 | HEL13 | 5 | 54°C | HEX | 176-194 |
| 20 | ILSTS05 | 5 | 56°C | FAM | 178-190 |
| 21 | INRA035 | 5 | 60°C | FAM | 99-123 |
| 22 | TGLA126 | 5 | 54°C | HEX | 114-128 |
| 23 | BM2113 | 6 | 63°C | FAM | 118-146 |
| 24 | ETH10 | 6 | 61°C | FAM | 207-225 |
| 25 | ETH225 | 6 | 63°C | ATTO550 | 140-162 |
| 26 | INRA023 | 6 | 58°C | ATTO550 | 199-219 |
| 27 | TGLA122 | 6 | 58°C | HEX | 134-174 |
