# Supplementary material for: Legacy of draught cattle breeds of South India: Insights into population structure, genetic admixture and maternal origin
Source: PLoS One. 2021 May 24;16(5):e0246497. doi: 10.1371/journal.pone.0246497 (PMC8143428; doi:10.1371/journal.pone.0246497)
Supplement: S2 Table — (DOCX) [file pone.0246497.s005.docx]

S2 Table. Accession numbers, population information and haplogroup details of GenBank sequences used in the study

| S.No | Country | Accession No. | Population  Name | Population Code | Population Details | Haplo  group |
| --- | --- | --- | --- | --- | --- | --- |
| 1 | Bhutan | AB268559 | Bhutanese native cattle | BUC | Native Bhutanese | I2 |
| 2 | Bhutan | AB268568 | Bhutanese native cattle | BUC | Native Bhutanese | I2 |
| 3 | Bhutan | AB570146 | Bhutanese native cattle | BUC | Native Bhutanese | I1 |
| 4 | Bhutan | AB570147 | Bhutanese native cattle | BUC | Native Bhutanese | I2 |
| 5 | Bhutan | AB570147 | Bhutanese native cattle | BUC | Native Bhutanese | I2 |
| 6 | Bhutan | AB570148 | Bhutanese native cattle | BUC | Native Bhutanese | I1 |
| 7 | Bhutan | AB570149 | Bhutanese native cattle | BUC | Native Bhutanese | I2 |
| 8 | Bhutan | AB570149 | Bhutanese native cattle | BUC | Native Bhutanese | I2 |
| 9 | Bhutan | AB570150 | Bhutanese native cattle | BUC | Native Bhutanese | I1 |
| 10 | Bhutan | AB570151 | Bhutanese native cattle | BUC | Native Bhutanese | I1 |
| 11 | Bhutan | AB570152 | Bhutanese native cattle | BUC | Native Bhutanese | I2 |
| 12 | Bhutan | AB570153 | Bhutanese native cattle | BUC | Native Bhutanese | I1 |
| 13 | Bhutan | AB268569 | Bhutanese native cattle | BUC | Native Bhutanese | I2 |
| 14 | Bhutan | AB570154 | Bhutanese native cattle | BUC | Native Bhutanese | I2 |
| 15 | Bhutan | AB570155 | Bhutanese native cattle | BUC | Native Bhutanese | I2 |
| 16 | Bhutan | AB570156 | Bhutanese native cattle | BUC | Native Bhutanese | I2 |
| 17 | Bhutan | AB570157 | Bhutanese native cattle | BUC | Native Bhutanese | I2 |
| 18 | Bhutan | AB570158 | Bhutanese native cattle | BUC | Native Bhutanese | I1 |
| 19 | Bhutan | AB570159 | Bhutanese native cattle | BUC | Native Bhutanese | T3 |
| 20 | Bhutan | AB570160 | Bhutanese native cattle | BUC | Native Bhutanese | I1 |
| 21 | Bhutan | AB570161 | Bhutanese native cattle | BUC | Native Bhutanese | I1 |
| 22 | Bhutan | AB570162 | Bhutanese native cattle | BUC | Native Bhutanese | I1 |
| 23 | Bhutan | AB570163 | Bhutanese native cattle | BUC | Native Bhutanese | T3 |
| 24 | Bhutan | AB268570 | Bhutanese native cattle | BUC | Native Bhutanese | I2 |
| 25 | Bhutan | AB570164 | Bhutanese native cattle | BUC | Native Bhutanese | T3 |
| 26 | Bhutan | AB570165 | Bhutanese native cattle | BUC | Native Bhutanese | I1 |
| 27 | Bhutan | AB268571 | Bhutanese native cattle | BUC | Native Bhutanese | I1 |
| 28 | Bhutan | AB268572 | Bhutanese native cattle | BUC | Native Bhutanese | I1 |
| 29 | Bhutan | AB268573 | Bhutanese native cattle | BUC | Native Bhutanese | I2 |
| 30 | Bhutan | AB268574 | Bhutanese native cattle | BUC | Native Bhutanese | I2 |
| 31 | Bhutan | AB268575 | Bhutanese native cattle | BUC | Native Bhutanese | I2 |
| 32 | Bhutan | AB268576 | Bhutanese native cattle | BUC | Native Bhutanese | I1 |
| 33 | Bhutan | AB268577 | Bhutanese native cattle | BUC | Native Bhutanese | I1 |
| 34 | Bhutan | AB268560 | Bhutanese native cattle | BUC | Native Bhutanese | I2 |
| 35 | Bhutan | AB268578 | Bhutanese native cattle | BUC | Native Bhutanese | I1 |
| 36 | Bhutan | AB268579 | Bhutanese native cattle | BUC | Native Bhutanese | I1 |
| 37 | Bhutan | AB268580 | Bhutanese native cattle | BUC | Native Bhutanese | I1 |
| 38 | Bhutan | AB268581 | Bhutanese native cattle | BUC | Native Bhutanese | I2 |
| 39 | Bhutan | AB570113 | Bhutanese native cattle | BUC | Native Bhutanese | I1 |
| 40 | Bhutan | AB570113 | Bhutanese native cattle | BUC | Native Bhutanese | I1 |
| 41 | Bhutan | AB570113 | Bhutanese native cattle | BUC | Native Bhutanese | I1 |
| 42 | Bhutan | AB570114 | Bhutanese native cattle | BUC | Native Bhutanese | I2 |
| 43 | Bhutan | AB570114 | Bhutanese native cattle | BUC | Native Bhutanese | I2 |
| 44 | Bhutan | AB570114 | Bhutanese native cattle | BUC | Native Bhutanese | I2 |
| 45 | Bhutan | AB268561 | Bhutanese native cattle | BUC | Native Bhutanese | I2 |
| 46 | Bhutan | AB570114 | Bhutanese native cattle | BUC | Native Bhutanese | I2 |
| 47 | Bhutan | AB570115 | Bhutanese native cattle | BUC | Native Bhutanese | I2 |
| 48 | Bhutan | AB570116 | Bhutanese native cattle | BUC | Native Bhutanese | I2 |
| 49 | Bhutan | AB570116 | Bhutanese native cattle | BUC | Native Bhutanese | I2 |
| 50 | Bhutan | AB570116 | Bhutanese native cattle | BUC | Native Bhutanese | I2 |
| 51 | Bhutan | AB570116 | Bhutanese native cattle | BUC | Native Bhutanese | I2 |
| 52 | Bhutan | AB570117 | Bhutanese native cattle | BUC | Native Bhutanese | I1 |
| 53 | Bhutan | AB570118 | Bhutanese native cattle | BUC | Native Bhutanese | I1 |
| 54 | Bhutan | AB570119 | Bhutanese native cattle | BUC | Native Bhutanese | I1 |
| 55 | Bhutan | AB570120 | Bhutanese native cattle | BUC | Native Bhutanese | I1 |
| 56 | Bhutan | AB268562 | Bhutanese native cattle | BUC | Native Bhutanese | I2 |
| 57 | Bhutan | AB570120 | Bhutanese native cattle | BUC | Native Bhutanese | I1 |
| 58 | Bhutan | AB570120 | Bhutanese native cattle | BUC | Native Bhutanese | I1 |
| 59 | Bhutan | AB570120 | Bhutanese native cattle | BUC | Native Bhutanese | I1 |
| 60 | Bhutan | AB570120 | Bhutanese native cattle | BUC | Native Bhutanese | I1 |
| 61 | Bhutan | AB570120 | Bhutanese native cattle | BUC | Native Bhutanese | I1 |
| 62 | Bhutan | AB570120 | Bhutanese native cattle | BUC | Native Bhutanese | I1 |
| 63 | Bhutan | AB570121 | Bhutanese native cattle | BUC | Native Bhutanese | I1 |
| 64 | Bhutan | AB570121 | Bhutanese native cattle | BUC | Native Bhutanese | I1 |
| 65 | Bhutan | AB570121 | Bhutanese native cattle | BUC | Native Bhutanese | I1 |
| 66 | Bhutan | AB570121 | Bhutanese native cattle | BUC | Native Bhutanese | I1 |
| 67 | Bhutan | AB268563 | Bhutanese native cattle | BUC | Native Bhutanese | I1 |
| 68 | Bhutan | AB570122 | Bhutanese native cattle | BUC | Native Bhutanese | I1 |
| 69 | Bhutan | AB570122 | Bhutanese native cattle | BUC | Native Bhutanese | I1 |
| 70 | Bhutan | AB570122 | Bhutanese native cattle | BUC | Native Bhutanese | I1 |
| 71 | Bhutan | AB570122 | Bhutanese native cattle | BUC | Native Bhutanese | I1 |
| 72 | Bhutan | AB570122 | Bhutanese native cattle | BUC | Native Bhutanese | I1 |
| 73 | Bhutan | AB570122 | Bhutanese native cattle | BUC | Native Bhutanese | I1 |
| 74 | Bhutan | AB570122 | Bhutanese native cattle | BUC | Native Bhutanese | I1 |
| 75 | Bhutan | AB570122 | Bhutanese native cattle | BUC | Native Bhutanese | I1 |
| 76 | Bhutan | AB570122 | Bhutanese native cattle | BUC | Native Bhutanese | I1 |
| 77 | Bhutan | AB570122 | Bhutanese native cattle | BUC | Native Bhutanese | I1 |
| 78 | Bhutan | AB268564 | Bhutanese native cattle | BUC | Native Bhutanese | I1 |
| 79 | Bhutan | AB570122 | Bhutanese native cattle | BUC | Native Bhutanese | I1 |
| 80 | Bhutan | AB570122 | Bhutanese native cattle | BUC | Native Bhutanese | I1 |
| 81 | Bhutan | AB570123 | Bhutanese native cattle | BUC | Native Bhutanese | I1 |
| 82 | Bhutan | AB570123 | Bhutanese native cattle | BUC | Native Bhutanese | I1 |
| 83 | Bhutan | AB570124 | Bhutanese native cattle | BUC | Native Bhutanese | T3 |
| 84 | Bhutan | AB570124 | Bhutanese native cattle | BUC | Native Bhutanese | T3 |
| 85 | Bhutan | AB570124 | Bhutanese native cattle | BUC | Native Bhutanese | T3 |
| 86 | Bhutan | AB570124 | Bhutanese native cattle | BUC | Native Bhutanese | T3 |
| 87 | Bhutan | AB570125 | Bhutanese native cattle | BUC | Native Bhutanese | I1 |
| 88 | Bhutan | AB570125 | Bhutanese native cattle | BUC | Native Bhutanese | I1 |
| 89 | Bhutan | AB268565 | Bhutanese native cattle | BUC | Native Bhutanese | I1 |
| 90 | Bhutan | AB570125 | Bhutanese native cattle | BUC | Native Bhutanese | I1 |
| 91 | Bhutan | AB570126 | Bhutanese native cattle | BUC | Native Bhutanese | I2 |
| 92 | Bhutan | AB570127 | Bhutanese native cattle | BUC | Native Bhutanese | I2 |
| 93 | Bhutan | AB570128 | Bhutanese native cattle | BUC | Native Bhutanese | T3 |
| 94 | Bhutan | AB570128 | Bhutanese native cattle | BUC | Native Bhutanese | T3 |
| 95 | Bhutan | AB570129 | Bhutanese native cattle | BUC | Native Bhutanese | T3 |
| 96 | Bhutan | AB570130 | Bhutanese native cattle | BUC | Native Bhutanese | T3 |
| 97 | Bhutan | AB570130 | Bhutanese native cattle | BUC | Native Bhutanese | T3 |
| 98 | Bhutan | AB570130 | Bhutanese native cattle | BUC | Native Bhutanese | T3 |
| 99 | Bhutan | AB570131 | Bhutanese native cattle | BUC | Native Bhutanese | I1 |
| 100 | Bhutan | AB268566 | Bhutanese native cattle | BUC | Native Bhutanese | I1 |
| 101 | Bhutan | AB570132 | Bhutanese native cattle | BUC | Native Bhutanese | I1 |
| 102 | Bhutan | AB570133 | Bhutanese native cattle | BUC | Native Bhutanese | I2 |
| 103 | Bhutan | AB570133 | Bhutanese native cattle | BUC | Native Bhutanese | I2 |
| 104 | Bhutan | AB570134 | Bhutanese native cattle | BUC | Native Bhutanese | I2 |
| 105 | Bhutan | AB570135 | Bhutanese native cattle | BUC | Native Bhutanese | I2 |
| 106 | Bhutan | AB570136 | Bhutanese native cattle | BUC | Native Bhutanese | I1 |
| 107 | Bhutan | AB570137 | Bhutanese native cattle | BUC | Native Bhutanese | T3 |
| 108 | Bhutan | AB570137 | Bhutanese native cattle | BUC | Native Bhutanese | T3 |
| 109 | Bhutan | AB570137 | Bhutanese native cattle | BUC | Native Bhutanese | T3 |
| 110 | Bhutan | AB570137 | Bhutanese native cattle | BUC | Native Bhutanese | T3 |
| 111 | Bhutan | AB268567 | Bhutanese native cattle | BUC | Native Bhutanese | I1 |
| 112 | Bhutan | AB570138 | Bhutanese native cattle | BUC | Native Bhutanese | T3 |
| 113 | Bhutan | AB570139 | Bhutanese native cattle | BUC | Native Bhutanese | I1 |
| 114 | Bhutan | AB570140 | Bhutanese native cattle | BUC | Native Bhutanese | I2 |
| 115 | Bhutan | AB570140 | Bhutanese native cattle | BUC | Native Bhutanese | I2 |
| 116 | Bhutan | AB570141 | Bhutanese native cattle | BUC | Native Bhutanese | I2 |
| 117 | Bhutan | AB570142 | Bhutanese native cattle | BUC | Native Bhutanese | I2 |
| 118 | Bhutan | AB570143 | Bhutanese native cattle | BUC | Native Bhutanese | I1 |
| 119 | Bhutan | AB570144 | Bhutanese native cattle | BUC | Native Bhutanese | I2 |
| 120 | Bhutan | AB570144 | Bhutanese native cattle | BUC | Native Bhutanese | I2 |
| 121 | Bhutan | AB570145 | Bhutanese native cattle | BUC | Native Bhutanese | T3 |
| 122 | China | EF417970 | Chinese cattle | CNC | Chinese-BF | I1 |
| 123 | China | EF417971 | Chinese cattle | CNC | Chinese-BF | I1 |
| 124 | China | EF417972 | Chinese cattle | CNC | Chinese-BM | I1 |
| 125 | China | EF417973 | Chinese cattle | CNC | Chinese-BM | I1 |
| 126 | China | EF417974 | Chinese cattle | CNC | Chinese-BM | I1 |
| 127 | China | EF417975 | Chinese cattle | CNC | Chinese-BM | I1 |
| 128 | China | EF417976 | Chinese cattle | CNC | Chinese-BM | I1 |
| 129 | China | EF417977 | Chinese cattle | CNC | Chinese-BM | I1 |
| 130 | China | DQ887760 | Chinese cattle | CNC | Chinese Brah | T3 |
| 131 | China | DQ887761 | Chinese cattle | CNC | Chinese Brah | T3 |
| 132 | China | DQ887762 | Chinese cattle | CNC | Chinese Brah | T3 |
| 133 | China | DQ887763 | Chinese cattle | CNC | Chinese Brah | T3 |
| 134 | China | DQ887764 | Chinese cattle | CNC | Chinese Brah | T3 |
| 135 | China | DQ887765 | Chinese cattle | CNC | Chinese Brah | I1 |
| 136 | China | DQ887766 | Chinese cattle | CNC | Chinese Brah | T1 |
| 137 | China | DQ887767 | Chinese cattle | CNC | Chinese Brah | T1 |
| 138 | China | DQ887768 | Chinese cattle | CNC | Chinese Brah | T1 |
| 139 | China | EF524165 | Chinese cattle | CNC | Chinese GF | I1 |
| 140 | China | EF524166 | Chinese cattle | CNC | Chinese GF | I1 |
| 141 | China | EF524167 | Chinese cattle | CNC | Chinese GF | I1 |
| 142 | China | EF524169 | Chinese cattle | CNC | Chinese GF | I1 |
| 143 | China | EF524170 | Chinese cattle | CNC | Chinese GF | I1 |
| 144 | China | EF524171 | Chinese cattle | CNC | Chinese GF | I1 |
| 145 | China | EF524172 | Chinese cattle | CNC | Chinese GF | I1 |
| 146 | China | EF524181 | Chinese cattle | CNC | Chinese PW | I1 |
| 147 | China | EF524177 | Chinese cattle | CNC | Chinese PW | I1 |
| 148 | China | EF524178 | Chinese cattle | CNC | Chinese PW | I1 |
| 149 | China | EF524180 | Chinese cattle | CNC | Chinese PW | I1 |
| 150 | China | EF524158 | Chinese cattle | CNC | Chinese RB | I1 |
| 151 | China | EF524160 | Chinese cattle | CNC | Chinese RB | I1 |
| 152 | China | EF524163 | Chinese cattle | CNC | Chinese RB | I1 |
| 153 | China | EF417978 | Chinese cattle | CNC | Chinese-RF | I1 |
| 154 | China | EF417979 | Chinese cattle | CNC | Chinese-RM | I1 |
| 155 | China | EF417980 | Chinese cattle | CNC | Chinese-RM | I1 |
| 156 | China | EF417981 | Chinese cattle | CNC | Chinese-RM | I1 |
| 157 | China | EF417982 | Chinese cattle | CNC | Chinese-RM | I1 |
| 158 | China | EF417983 | Chinese cattle | CNC | Chinese-RM | I1 |
| 159 | China | EF417984 | Chinese cattle | CNC | Chinese-RM | I1 |
| 160 | China | EF417985 | Chinese cattle | CNC | Chinese-RM | I1 |
| 161 | China | EF417986 | Chinese cattle | CNC | Chinese-RM | I1 |
| 162 | China | EF524151 | Chinese cattle | CNC | Chinese TC | I1 |
| 163 | China | EF524152 | Chinese cattle | CNC | Chinese TC | I1 |
| 164 | China | EF524156 | Chinese cattle | CNC | Chinese TC | I1 |
| 165 | China | EF524182 | Chinese cattle | CNC | Chinese XX | I1 |
| 166 | China | EF524183 | Chinese cattle | CNC | Chinese XX | I1 |
| 167 | China | EF524184 | Chinese cattle | CNC | Chinese XX | I1 |
| 168 | China | EF524185 | Chinese cattle | CNC | Chinese XX | I1 |
| 169 | India | KP223257 | North Indian cattle | NIC | Bachaur | I2 |
| 170 | India | KP223258 | North Indian cattle | NIC | Bachaur | I1 |
| 171 | India | KP223259 | North Indian cattle | NIC | Bachaur | I1 |
| 172 | India | KP223260 | North Indian cattle | NIC | Bachaur | I1 |
| 173 | India | KP223261 | North Indian cattle | NIC | Gangatiri | I1 |
| 174 | India | KP223262 | North Indian cattle | NIC | Gangatiri | I1 |
| 175 | India | KP223263 | North Indian cattle | NIC | Gangatiri | I1 |
| 176 | India | KP223264 | North Indian cattle | NIC | Gangatiri | T3 |
| 177 | India | KP223265 | North Indian cattle | NIC | Kenkatha | T2 |
| 178 | India | KP223266 | North Indian cattle | NIC | Kenkatha | I2 |
| 179 | India | KP223267 | North Indian cattle | NIC | Kenkatha | I1 |
| 180 | India | KP223268 | North Indian cattle | NIC | Kenkatha | I2 |
| 181 | India | KP223269 | North Indian cattle | NIC | Kherigarh | I2 |
| 182 | India | KP223270 | North Indian cattle | NIC | Kherigarh | I2 |
| 183 | India | KP223271 | North Indian cattle | NIC | Kherigarh | I2 |
| 184 | India | KP223272 | North Indian cattle | NIC | Kherigarh | T3 |
| 185 | India | GQ890097 | North Indian cattle | NIC | Malvi | I2 |
| 186 | India | GQ890106 | North Indian cattle | NIC | Malvi | I1 |
| 187 | India | GQ890107 | North Indian cattle | NIC | Malvi | I1 |
| 188 | India | GQ890108 | North Indian cattle | NIC | Malvi | I2 |
| 189 | India | GQ890098 | North Indian cattle | NIC | Malvi | I1 |
| 190 | India | GQ890099 | North Indian cattle | NIC | Malvi | I2 |
| 191 | India | GQ890100 | North Indian cattle | NIC | Malvi | I1 |
| 192 | India | GQ890101 | North Indian cattle | NIC | Malvi | I1 |
| 193 | India | GQ890102 | North Indian cattle | NIC | Malvi | I2 |
| 194 | India | GQ890103 | North Indian cattle | NIC | Malvi | I1 |
| 195 | India | GQ890104 | North Indian cattle | NIC | Malvi | I1 |
| 196 | India | GQ890105 | North Indian cattle | NIC | Malvi | I1 |
| 197 | India | GQ890083 | North Indian cattle | NIC | Mewati | T3 |
| 198 | India | GQ890092 | North Indian cattle | NIC | Mewati | I1 |
| 199 | India | GQ890084 | North Indian cattle | NIC | Mewati | I1 |
| 200 | India | GQ890085 | North Indian cattle | NIC | Mewati | I2 |
| 201 | India | GQ890086 | North Indian cattle | NIC | Mewati | I1 |
| 202 | India | GQ890087 | North Indian cattle | NIC | Mewati | I1 |
| 203 | India | GQ890088 | North Indian cattle | NIC | Mewati | I1 |
| 204 | India | GQ890089 | North Indian cattle | NIC | Mewati | I1 |
| 205 | India | GQ890090 | North Indian cattle | NIC | Mewati | I1 |
| 206 | India | GQ890091 | North Indian cattle | NIC | Mewati | I2 |
| 207 | India | GQ890096 | North Indian cattle | NIC | Nimari | I2 |
| 208 | India | GQ890109 | North Indian cattle | NIC | Nimari | I1 |
| 209 | India | GQ890110 | North Indian cattle | NIC | Nimari | I2 |
| 210 | India | GQ890111 | North Indian cattle | NIC | Nimari | I1 |
| 211 | India | GQ890112 | North Indian cattle | NIC | Nimari | I1 |
| 212 | India | KP223273 | North Indian cattle | NIC | Purnea | I1 |
| 213 | India | KP223274 | North Indian cattle | NIC | Purnea | I2 |
| 214 | India | KP223275 | North Indian cattle | NIC | Purnea | I1 |
| 215 | India | KP223276 | North Indian cattle | NIC | Purnea | T2 |
| 216 | India | KP223277 | North Indian cattle | NIC | Purnea | I1 |
| 217 | India | KP223278 | North Indian cattle | NIC | Purnea | I2 |
| 218 | India | KP223279 | North Indian cattle | NIC | Shahabadi | I2 |
| 219 | India | KP223280 | North Indian cattle | NIC | Shahabadi | I1 |
| 220 | India | KP223281 | North Indian cattle | NIC | Shahabadi | T2 |
| 221 | India | KP223282 | North Indian cattle | NIC | Shahabadi | I1 |
| 222 | Nepal | AB085922 | Nepalese native cattle | NPC | Hariana | I2 |
| 223 | Nepal | AB085923 | Nepalese native cattle | NPC | Hariana | I1 |
| 224 | Nepal | AB085921 | Nepalese native cattle | NPC | Lulu | I2 |
| 225 | Nepal | AB570166 | Nepalese native cattle | NPC | Native Nepalese | T3 |
| 226 | Nepal | AB570174 | Nepalese native cattle | NPC | Native Nepalese | I2 |
| 227 | Nepal | AB570175 | Nepalese native cattle | NPC | Native Nepalese | I2 |
| 228 | Nepal | AB570167 | Nepalese native cattle | NPC | Native Nepalese | I2 |
| 229 | Nepal | AB570168 | Nepalese native cattle | NPC | Native Nepalese | I1 |
| 230 | Nepal | AB570169 | Nepalese native cattle | NPC | Native Nepalese | I2 |
| 231 | Nepal | AB570170 | Nepalese native cattle | NPC | Native Nepalese | I1 |
| 232 | Nepal | AB570171 | Nepalese native cattle | NPC | Native Nepalese | I2 |
| 233 | Nepal | AB570172 | Nepalese native cattle | NPC | Native Nepalese | T3 |
| 234 | Nepal | AB570172 | Nepalese native cattle | NPC | Native Nepalese | T3 |
| 235 | Nepal | AB570173 | Nepalese native cattle | NPC | Native Nepalese | I1 |
| 236 | Vietnam | JX040458 | Vietnamese native cattle | VNC | LangSon | I1 |
| 237 | Vietnam | JX040467 | Vietnamese native cattle | VNC | LangSon | I1 |
| 238 | Vietnam | JX040468 | Vietnamese native cattle | VNC | LangSon | I1 |
| 239 | Vietnam | JX040469 | Vietnamese native cattle | VNC | LangSon | I1 |
| 240 | Vietnam | JX040459 | Vietnamese native cattle | VNC | LangSon | I1 |
| 241 | Vietnam | JX040460 | Vietnamese native cattle | VNC | LangSon | I1 |
| 242 | Vietnam | JX040461 | Vietnamese native cattle | VNC | LangSon | I1 |
| 243 | Vietnam | JX040462 | Vietnamese native cattle | VNC | LangSon | I1 |
| 244 | Vietnam | JX040463 | Vietnamese native cattle | VNC | LangSon | I1 |
| 245 | Vietnam | JX040464 | Vietnamese native cattle | VNC | LangSon | I1 |
| 246 | Vietnam | JX040465 | Vietnamese native cattle | VNC | LangSon | I1 |
| 247 | Vietnam | JX040466 | Vietnamese native cattle | VNC | LangSon | I1 |
| 248 | Vietnam | JX040452 | Vietnamese native cattle | VNC | ThanhHoa | I1 |
| 249 | Vietnam | JX040453 | Vietnamese native cattle | VNC | ThanhHoa | I1 |
| 250 | Vietnam | JX040454 | Vietnamese native cattle | VNC | ThanhHoa | I1 |
| 251 | Vietnam | JX040455 | Vietnamese native cattle | VNC | ThanhHoa | I1 |
| 252 | Vietnam | JX040456 | Vietnamese native cattle | VNC | ThanhHoa | I1 |
| 253 | Vietnam | JX040457 | Vietnamese native cattle | VNC | ThanhHoa | I1 |
| 254 | India | GQ890120 | West Indian cattle | WIC | Rathi | I1 |
| 255 | India | GQ890122 | West Indian cattle | WIC | Rathi | I2 |
| 256 | India | GQ890123 | West Indian cattle | WIC | Rathi | I1 |
| 257 | India | GQ890124 | West Indian cattle | WIC | Rathi | I1 |
| 258 | India | GQ890125 | West Indian cattle | WIC | Rathi | I1 |
| 259 | India | GQ890126 | West Indian cattle | WIC | Rathi | I1 |
| 260 | India | GQ890127 | West Indian cattle | WIC | Rathi | I2 |
| 261 | India | HQ234718 | West Indian cattle | WIC | RedSindhi | I1 |
| 262 | India | HQ234727 | West Indian cattle | WIC | RedSindhi | I1 |
| 263 | India | HQ234728 | West Indian cattle | WIC | RedSindhi | I2 |
| 264 | India | HQ234729 | West Indian cattle | WIC | RedSindhi | I1 |
| 265 | India | HQ234730 | West Indian cattle | WIC | RedSindhi | I2 |
| 266 | India | HQ234719 | West Indian cattle | WIC | RedSindhi | I1 |
| 267 | India | GQ890116 | West Indian cattle | WIC | Red Sindhi | I1 |
| 268 | India | GQ890121 | West Indian cattle | WIC | Red Sindhi | I1 |
| 269 | India | GQ890135 | West Indian cattle | WIC | Red Sindhi | I1 |
| 270 | India | HQ234720 | West Indian cattle | WIC | RedSindhi | I1 |
| 271 | India | HQ234721 | West Indian cattle | WIC | RedSindhi | I2 |
| 272 | India | HQ234722 | West Indian cattle | WIC | RedSindhi | I2 |
| 273 | India | HQ234723 | West Indian cattle | WIC | RedSindhi | I1 |
| 274 | India | HQ234724 | West Indian cattle | WIC | RedSindhi | I1 |
| 275 | India | HQ234725 | West Indian cattle | WIC | RedSindhi | I2 |
| 276 | India | HQ234726 | West Indian cattle | WIC | RedSindhi | I1 |
| 277 | India | AY378137 | West Indian cattle | WIC | Tharparkar | I1 |
| 278 | India | HQ234739 | West Indian cattle | WIC | Tharparkar | I1 |
| 279 | India | HQ234740 | West Indian cattle | WIC | Tharparkar | I1 |
| 280 | India | HQ234741 | West Indian cattle | WIC | Tharparkar | I1 |
| 281 | India | HQ234742 | West Indian cattle | WIC | Tharparkar | I1 |
| 282 | India | HQ234743 | West Indian cattle | WIC | Tharparkar | I1 |
| 283 | India | HQ234731 | West Indian cattle | WIC | Tharparkar | T3 |
| 284 | India | GQ890137 | West Indian cattle | WIC | Tharparkar | I2 |
| 285 | India | GQ890138 | West Indian cattle | WIC | Tharparkar | T3 |
| 286 | India | GQ890139 | West Indian cattle | WIC | Tharparkar | I1 |
| 287 | India | GQ890140 | West Indian cattle | WIC | Tharparkar | I1 |
| 288 | India | GQ890141 | West Indian cattle | WIC | Tharparkar | T3 |
| 289 | India | GQ890142 | West Indian cattle | WIC | Tharparkar | I1 |
| 290 | India | GQ890143 | West Indian cattle | WIC | Tharparkar | I1 |
| 291 | India | GQ890144 | West Indian cattle | WIC | Tharparkar | T3 |
| 292 | India | HQ234732 | West Indian cattle | WIC | Tharparkar | T3 |
| 293 | India | HQ234733 | West Indian cattle | WIC | Tharparkar | I1 |
| 294 | India | HQ234734 | West Indian cattle | WIC | Tharparkar | I1 |
| 295 | India | HQ234735 | West Indian cattle | WIC | Tharparkar | I1 |
| 296 | India | HQ234736 | West Indian cattle | WIC | Tharparkar | I1 |
| 297 | India | HQ234737 | West Indian cattle | WIC | Tharparkar | I2 |
| 298 | India | HQ234738 | West Indian cattle | WIC | Tharparkar | I2 |
