# Supplementary material for: Legacy of draught cattle breeds of South India: Insights into population structure, genetic admixture and maternal origin
Source: PLoS One. 2021 May 24;16(5):e0246497. doi: 10.1371/journal.pone.0246497 (PMC8143428; doi:10.1371/journal.pone.0246497)
Supplement: S3 Table — (DOCX) [file pone.0246497.s006.docx]

S3 Table. Comparison of diversity measures (mean and range) among Indian indigenous cattle across different regions.

| Diversity Parameter | South | North | West-Central | East |
| --- | --- | --- | --- | --- |
| Observed no.  alleles (n_a_) | 6.867 (5.926+8.074) | 8.784  (6.571-10.619) | 8.392  (8.905-10.619) | 9.486  (7.682-9.409) |
| Observed heterozygosity (H_o_) | 0.644  (0.598+0.687) | 0.653  (0.459-0.724) | 0.516  (0.681-0.721) | 0.704  (0.382-0.619) |
| Expected heterozygosity (H_e_) | 0.691 (0.637+0.727) | 0.685  (0.514-0.735) | 0.691  (0.702-0.751) | 0.720  (0.614-0.749) |
| Heterozygosity Deficit (F_IS_) | 0.069 (0.027+0.108) | 0.048  (-0.028-0.221) | 0.253  (-0.010-0.042) | 0.033  (0.0989-0.462) |
