# Supplementary material for: Legacy of draught cattle breeds of South India: Insights into population structure, genetic admixture and maternal origin
Source: PLoS One. 2021 May 24;16(5):e0246497. doi: 10.1371/journal.pone.0246497 (PMC8143428; doi:10.1371/journal.pone.0246497)
Supplement: S4 Table. Global F statistics among South Indian cattle (a) draught type zebu, taurine and crossbred cattle and (b) draught type zebu cattle only — (DOCX) [file pone.0246497.s007.docx]

S4 Table. Global F statistics among South Indian cattle (a) draught type zebu, taurine and crossbred cattle and (b) draught type zebu cattle only

| Locus | Zebu, taurine and crossbred cattle | | | Zebu cattle only | | |
| --- | --- | --- | --- | --- | --- | --- |
|  | F_IT_ | F_ST_ | F_IS_ | F_IT_ | F_ST_ | F_IS_ |
| CSRM60 | 0.145 | 0.070 | 0.080 | 0.150 | 0.045 | 0.110 |
| CSSM66 | 0.073 | 0.085 | -0.013 | 0.046 | 0.040 | 0.006 |
| HEL1 | 0.203 | 0.131 | 0.083 | 0.145 | 0.054 | 0.097 |
| INRA63 | 0.130 | 0.139 | -0.009 | 0.005 | 0.049 | -0.046 |
| BM1824 | 0.036 | 0.062 | -0.027 | 0.036 | 0.065 | -0.031 |
| ETH152 | 0.225 | 0.223 | 0.002 | 0.027 | 0.054 | -0.029 |
| HAUT27 | 0.318 | 0.099 | 0.243 | 0.370 | 0.068 | 0.323 |
| INRA05 | 0.113 | 0.052 | 0.064 | 0.101 | 0.024 | 0.079 |
| BM1818 | 0.097 | 0.074 | 0.025 | 0.100 | 0.046 | 0.056 |
| ETH3 | 0.148 | 0.126 | 0.025 | 0.088 | 0.050 | 0.040 |
| HEL9 | 0.156 | 0.111 | 0.051 | 0.117 | 0.067 | 0.053 |
| ILSTS006 | 0.124 | 0.069 | 0.059 | 0.150 | 0.060 | 0.096 |
| TGLA53 | 0.190 | 0.069 | 0.129 | 0.218 | 0.076 | 0.154 |
| HAUT24 | 0.299 | 0.124 | 0.201 | 0.327 | 0.114 | 0.242 |
| INRA032 | 0.127 | 0.093 | 0.037 | 0.092 | 0.046 | 0.048 |
| SPS115 | 0.115 | 0.090 | 0.028 | 0.113 | 0.073 | 0.044 |
| ETH185 | 0.143 | 0.108 | 0.039 | 0.103 | 0.069 | 0.037 |
| ILSTS05 | 0.066 | 0.090 | -0.027 | 0.007 | 0.043 | -0.037 |
| INRA035 | 0.286 | 0.163 | 0.147 | 0.194 | 0.084 | 0.121 |
| TGLA126 | 0.077 | 0.090 | -0.014 | 0.008 | 0.043 | -0.037 |
| BM2113 | 0.135 | 0.093 | 0.046 | 0.103 | 0.048 | 0.057 |
| ETH10 | 0.140 | 0.110 | 0.034 | 0.087 | 0.052 | 0.038 |
| ETH225 | 0.145 | 0.134 | 0.013 | 0.077 | 0.064 | 0.014 |
| INRA023 | 0.080 | 0.077 | 0.003 | 0.019 | 0.036 | -0.017 |
| TGLA122 | 0.064 | 0.083 | -0.021 | 0.027 | 0.058 | -0.033 |
| Overall | 0.143 | 0.101 | 0.047 | 0.109 | 0.057 | 0.056 |
