# Supplementary material for: Legacy of draught cattle breeds of South India: Insights into population structure, genetic admixture and maternal origin
Source: PLoS One. 2021 May 24;16(5):e0246497. doi: 10.1371/journal.pone.0246497 (PMC8143428; doi:10.1371/journal.pone.0246497)
Supplement: S5 Table — (DOCX) [file pone.0246497.s008.docx]

S5 Table. Frequency of mtDNA haplotypes and their sharing among different breeds of zebu, taurine and crossbred cattle

| Haplotype_ID | HFX | JRX | ALA | BAR | DEO |  | HAL | KAN | ONG | PUL | PNG | UMB | VEC | HFP | JER |
| --- | --- | --- | --- | --- | --- | --- | --- | --- | --- | --- | --- | --- | --- | --- | --- |
| >Hx_H1_T3 | 0.026 |  |  |  |  |  |  |  |  |  |  |  |  |  |  |
| >HxPHJ_H2_T3 | 0.026 |  |  |  |  |  |  |  |  | 0.032 |  |  |  | 0.067 | 0.091 |
| >Hx_H3_I1 | 0.026 |  |  |  |  |  |  |  |  |  |  |  |  |  |  |
| >HxD_H4_I1 | 0.026 |  |  |  | 0.038 |  |  |  |  |  |  |  |  |  |  |
| >Hx_H5_I2 | 0.026 |  |  |  |  |  |  |  |  |  |  |  |  |  |  |
| >Hx_H6_I2 | 0.051 |  |  |  |  |  |  |  |  |  |  |  |  |  |  |
| >HxJxABDLKOPUV_H7_I2 | 0.103 | 0.069 | 0.069 | 0.058 | 0.019 |  | 0.056 | 0.078 | 0.078 | 0.065 |  | 0.061 | 0.115 |  |  |
| >Hx_H8_I2 | 0.026 |  |  |  |  |  |  |  |  |  |  |  |  |  |  |
| >Hx_H9_I2 | 0.051 |  |  |  |  |  |  |  |  |  |  |  |  |  |  |
| >Hx_H10_I2 | 0.026 |  |  |  |  |  |  |  |  |  |  |  |  |  |  |
| >HxONU_H11_I2 | 0.026 |  |  |  |  |  |  |  | 0.039 |  | 0.063 | 0.061 |  |  |  |
| >HxJxDKOPU_H12_I2 | 0.026 | 0.052 |  |  | 0.038 |  |  | 0.078 | 0.020 | 0.032 |  | 0.121 |  |  |  |
| >HxJxABDLKOPUVHJ_H13_I1 | 0.154 | 0.259 | 0.103 | 0.212 | 0.189 |  | 0.111 | 0.216 | 0.255 | 0.097 |  | 0.152 | 0.039 | 0.333 | 0.030 |
| >HxJxADK_H14_I1 | 0.026 | 0.017 | 0.035 |  | 0.019 |  |  | 0.039 |  |  |  |  |  |  |  |
| >Hx_H15_I1 | 0.026 |  |  |  |  |  |  |  |  |  |  |  |  |  |  |
| >HxH_H16_I1 | 0.051 |  |  |  |  |  |  |  |  |  |  |  |  | 0.067 |  |
| >Hx_H17_I1 | 0.026 |  |  |  |  |  |  |  |  |  |  |  |  |  |  |
| >Hx_H18_I1 | 0.026 |  |  |  |  |  |  |  |  |  |  |  |  |  |  |
| >Hx_H19_I1 | 0.026 |  |  |  |  |  |  |  |  |  |  |  |  |  |  |
| >Hx_H20_I1 | 0.026 |  |  |  |  |  |  |  |  |  |  |  |  |  |  |
| >Hx_H21_I1 | 0.077 |  |  |  |  |  |  |  |  |  |  |  |  |  |  |
| >Hx_H22_I1 | 0.026 |  |  |  |  |  |  |  |  |  |  |  |  |  |  |
| >Hx_H23_I1 | 0.026 |  |  |  |  |  |  |  |  |  |  |  |  |  |  |
| >Hx_H24_I1 | 0.026 |  |  |  |  |  |  |  |  |  |  |  |  |  |  |
| >HxALOU_H25_I1 | 0.026 |  | 0.069 |  |  |  | 0.028 |  | 0.020 |  |  | 0.061 |  |  |  |
| >Hx_H26_I1 | 0.026 |  |  |  |  |  |  |  |  |  |  |  |  |  |  |
| >JxB_H27_I1 |  | 0.017 |  | 0.039 |  |  |  |  |  |  |  |  |  |  |  |
| >JxAP_H28_I1 |  | 0.017 | 0.035 |  |  |  |  |  |  | 0.032 |  |  |  |  |  |
| >Jx_H29_I1 |  | 0.017 |  |  |  |  |  |  |  |  |  |  |  |  |  |
| >Jx_H30_I1 |  | 0.017 |  |  |  |  |  |  |  |  |  |  |  |  |  |
| >Jx_H31_I1 |  | 0.017 |  |  |  |  |  |  |  |  |  |  |  |  |  |
| >JxABKOPU_H32_I1 |  | 0.035 | 0.103 | 0.039 |  |  |  | 0.039 | 0.020 | 0.065 |  | 0.030 |  |  |  |
| >Jx_H33_I1 |  | 0.017 |  |  |  |  |  |  |  |  |  |  |  |  |  |
| >Jx_H34_I1 |  | 0.035 |  |  |  |  |  |  |  |  |  |  |  |  |  |
| >Jx_H35_I1 |  | 0.017 |  |  |  |  |  |  |  |  |  |  |  |  |  |
| >Jx_H36_I1 |  | 0.017 |  |  |  |  |  |  |  |  |  |  |  |  |  |
| >Jx_H37_I1 |  | 0.017 |  |  |  |  |  |  |  |  |  |  |  |  |  |
| >Jx_H38_I1 |  | 0.017 |  |  |  |  |  |  |  |  |  |  |  |  |  |
| >JxABDLKOPNUVH_H39_I1 |  | 0.017 | 0.035 | 0.019 | 0.094 |  | 0.083 | 0.020 | 0.059 | 0.065 | 0.562 | 0.030 | 0.039 | 0.067 |  |
| >JxAU_H40_I1 |  | 0.017 | 0.035 |  |  |  |  |  |  |  |  | 0.030 |  |  |  |
| >Jx_H41_I1 |  | 0.017 |  |  |  |  |  |  |  |  |  |  |  |  |  |
| >Jx_H42_I1 |  | 0.017 |  |  |  |  |  |  |  |  |  |  |  |  |  |
| >Jx_H43_I1 |  | 0.017 |  |  |  |  |  |  |  |  |  |  |  |  |  |
| >JxJL_H44_I1 |  | 0.017 |  |  |  |  | 0.028 |  | 0.039 |  |  |  |  |  |  |
| >Jx_H45_I1 |  | 0.017 |  |  |  |  |  |  |  |  |  |  |  |  |  |
| >JxL_H46_I1 |  | 0.017 |  |  |  |  | 0.028 |  |  |  |  |  |  |  |  |
| >Jx_H47_I2 |  | 0.017 |  |  |  |  |  |  |  |  |  |  |  |  |  |
| >Jx_H48_I2 |  | 0.017 |  |  |  |  |  |  |  |  |  |  |  |  |  |
| >Jx_H49_I2 |  | 0.017 |  |  |  |  |  |  |  |  |  |  |  |  |  |
| >Jx_H50_I2 |  | 0.017 |  |  |  |  |  |  |  |  |  |  |  |  |  |
| >Jx_H51_I2 |  | 0.017 |  |  |  |  |  |  |  |  |  |  |  |  |  |
| >Jx_H52_I2 |  | 0.017 |  |  |  |  |  |  |  |  |  |  |  |  |  |
| >Jx_H53_I2 |  | 0.017 |  |  |  |  |  |  |  |  |  |  |  |  |  |
| >Jx_H54_I2 |  | 0.017 |  |  |  |  |  |  |  |  |  |  |  |  |  |
| >Jx_H55_I2 |  | 0.017 |  |  |  |  |  |  |  |  |  |  |  |  |  |
| >Jx_H56_I2 |  | 0.017 |  |  |  |  |  |  |  |  |  |  |  |  |  |
| >Jx_H57_I2 |  | 0.017 |  |  |  |  |  |  |  |  |  |  |  |  |  |
| >JxB_H58_I2 |  | 0.017 |  | 0.039 |  |  |  |  |  |  |  |  |  |  |  |
| >Jx_H59_I2 |  | 0.017 |  |  |  |  |  |  |  |  |  |  |  |  |  |
| >A_H60_I2 |  |  | 0.035 |  |  |  |  |  |  |  |  |  |  |  |  |
| >AD_H61_I2 |  |  | 0.035 |  | 0.019 |  |  |  |  |  |  |  |  |  |  |
| >A_H62_I2 |  |  | 0.035 |  |  |  |  |  |  |  |  |  |  |  |  |
| >A_H63_I2 |  |  | 0.035 |  |  |  |  |  |  |  |  |  |  |  |  |
| >ABU_H64_I2 |  |  | 0.069 | 0.039 |  |  |  |  |  |  |  | 0.030 |  |  |  |
| >A_H65_I1 |  |  | 0.035 |  |  |  |  |  |  |  |  |  |  |  |  |
| >A_H66_I1 |  |  | 0.035 |  |  |  |  |  |  |  |  |  |  |  |  |
| >ABOH_H67_I1 |  |  | 0.035 | 0.019 |  |  |  |  | 0.039 |  |  |  |  | 0.067 |  |
| >AU_H68_I1 |  |  | 0.035 |  |  |  |  |  |  |  |  | 0.030 |  |  |  |
| >A_H69_I1 |  |  | 0.035 |  |  |  |  |  |  |  |  |  |  |  |  |
| >A_H70_I1 |  |  | 0.103 |  |  |  |  |  |  |  |  |  |  |  |  |
| >A_H71_I1 |  |  | 0.035 |  |  |  |  |  |  |  |  |  |  |  |  |
| >B_H72_T2 |  |  |  | 0.019 |  |  |  |  |  |  |  |  |  |  |  |
| >B_H73_I1 |  |  |  | 0.019 |  |  |  |  |  |  |  |  |  |  |  |
| >B_H74_I1 |  |  |  | 0.058 |  |  |  |  |  |  |  |  |  |  |  |
| >B_H75_I1 |  |  |  | 0.058 |  |  |  |  |  |  |  |  |  |  |  |
| >B_H76_I1 |  |  |  | 0.019 |  |  |  |  |  |  |  |  |  |  |  |
| >B_H77_I1 |  |  |  | 0.019 |  |  |  |  |  |  |  |  |  |  |  |
| >B_H78_I1 |  |  |  | 0.019 |  |  |  |  |  |  |  |  |  |  |  |
| >B_H79_I1 |  |  |  | 0.019 |  |  |  |  |  |  |  |  |  |  |  |
| >B_H80_I1 |  |  |  | 0.019 |  |  |  |  |  |  |  |  |  |  |  |
| >B_H81_I1 |  |  |  | 0.058 |  |  |  |  |  |  |  |  |  |  |  |
| >BU_H82_I1 |  |  |  | 0.019 |  |  |  |  |  |  |  | 0.061 |  |  |  |
| >BL_H83_I1 |  |  |  | 0.019 |  |  | 0.028 |  |  |  |  |  |  |  |  |
| >B_H84_I2 |  |  |  | 0.039 |  |  |  |  |  |  |  |  |  |  |  |
| >B_H85_I2 |  |  |  | 0.019 |  |  |  |  |  |  |  |  |  |  |  |
| >B_H86_I2 |  |  |  | 0.077 |  |  |  |  |  |  |  |  |  |  |  |
| >B_H87_I2 |  |  |  | 0.058 |  |  |  |  |  |  |  |  |  |  |  |
| >D_H88_T1 |  |  |  |  | 0.019 |  |  |  |  |  |  |  |  |  |  |
| >D_H89_T3 |  |  |  |  | 0.019 |  |  |  |  |  |  |  |  |  |  |
| >D_H90_I2 |  |  |  |  | 0.019 |  |  |  |  |  |  |  |  |  |  |
| >D_H91_I2 |  |  |  |  | 0.019 |  |  |  |  |  |  |  |  |  |  |
| >D_H92_I2 |  |  |  |  | 0.019 |  |  |  |  |  |  |  |  |  |  |
| >D_H93_I2 |  |  |  |  | 0.038 |  |  |  |  |  |  |  |  |  |  |
| >D_H94_I2 |  |  |  |  | 0.019 |  |  |  |  |  |  |  |  |  |  |
| >D_H95_I2 |  |  |  |  | 0.019 |  |  |  |  |  |  |  |  |  |  |
| >D_H96_I2 |  |  |  |  | 0.019 |  |  |  |  |  |  |  |  |  |  |
| >D_H97_I1 |  |  |  |  | 0.038 |  |  |  |  |  |  |  |  |  |  |
| >D_H98_I1 |  |  |  |  | 0.019 |  |  |  |  |  |  |  |  |  |  |
| >D_H99_I1 |  |  |  |  | 0.019 |  |  |  |  |  |  |  |  |  |  |
| >D_H100_I1 |  |  |  |  | 0.019 |  |  |  |  |  |  |  |  |  |  |
| >D_H101_I1 |  |  |  |  | 0.019 |  |  |  |  |  |  |  |  |  |  |
| >D_H102_I1 |  |  |  |  | 0.038 |  |  |  |  |  |  |  |  |  |  |
| >D_H103_I1 |  |  |  |  | 0.019 |  |  |  |  |  |  |  |  |  |  |
| >D_H104_I1 |  |  |  |  | 0.038 |  |  |  |  |  |  |  |  |  |  |
| >DK_H105_I1 |  |  |  |  | 0.038 |  |  | 0.255 |  |  |  |  |  |  |  |
| >D_H106_I1 |  |  |  |  | 0.038 |  |  |  |  |  |  |  |  |  |  |
| >D_H107_I1 |  |  |  |  | 0.019 |  |  |  |  |  |  |  |  |  |  |
| >D_H108_I1 |  |  |  |  | 0.057 |  |  |  |  |  |  |  |  |  |  |
| >D_H109_I1 |  |  |  |  | 0.019 |  |  |  |  |  |  |  |  |  |  |
| >D_H110_I1 |  |  |  |  | 0.019 |  |  |  |  |  |  |  |  |  |  |
| >L_H111_T2 |  |  |  |  |  |  | 0.028 |  |  |  |  |  |  |  |  |
| >L_H112_I1 |  |  |  |  |  |  | 0.028 |  |  |  |  |  |  |  |  |
| >L_H113_I1 |  |  |  |  |  |  | 0.056 |  |  |  |  |  |  |  |  |
| >L_H114_I1 |  |  |  |  |  |  | 0.028 |  |  |  |  |  |  |  |  |
| >L_H115_I1 |  |  |  |  |  |  | 0.028 |  |  |  |  |  |  |  |  |
| >L_H116_I2 |  |  |  |  |  |  | 0.028 |  |  |  |  |  |  |  |  |
| >LU_H117_I1 |  |  |  |  |  |  | 0.028 |  |  |  |  | 0.030 |  |  |  |
| >L_H118_I2 |  |  |  |  |  |  | 0.028 |  |  |  |  |  |  |  |  |
| >L_H119_I2 |  |  |  |  |  |  | 0.028 |  |  |  |  |  |  |  |  |
| >L_H120_I1 |  |  |  |  |  |  | 0.028 |  |  |  |  |  |  |  |  |
| >L_H121_I1 |  |  |  |  |  |  | 0.028 |  |  |  |  |  |  |  |  |
| >L_H122_I2 |  |  |  |  |  |  | 0.028 |  |  |  |  |  |  |  |  |
| >L_H123_I1 |  |  |  |  |  |  | 0.028 |  |  |  |  |  |  |  |  |
| >L_H124_I1 |  |  |  |  |  |  | 0.028 |  |  |  |  |  |  |  |  |
| >L_H125_I1 |  |  |  |  |  |  | 0.028 |  |  |  |  |  |  |  |  |
| >L_H126_I2 |  |  |  |  |  |  | 0.028 |  |  |  |  |  |  |  |  |
| >L_H127_I2 |  |  |  |  |  |  | 0.028 |  |  |  |  |  |  |  |  |
| >LNV_H128_I2 |  |  |  |  |  |  | 0.056 |  |  |  | 0.063 |  | 0.077 |  |  |
| >L_H129_I2 |  |  |  |  |  |  | 0.028 |  |  |  |  |  |  |  |  |
| >L_H130_I2 |  |  |  |  |  |  | 0.028 |  |  |  |  |  |  |  |  |
| >L_H131_I2 |  |  |  |  |  |  | 0.028 |  |  |  |  |  |  |  |  |
| >K_H132_T2 |  |  |  |  |  |  |  | 0.020 |  |  |  |  |  |  |  |
| >K_H133_I2 |  |  |  |  |  |  |  | 0.059 |  |  |  |  |  |  |  |
| >K_H134_I2 |  |  |  |  |  |  |  | 0.020 |  |  |  |  |  |  |  |
| >K_H135_I2 |  |  |  |  |  |  |  | 0.020 |  |  |  |  |  |  |  |
| >K_H136_I2 |  |  |  |  |  |  |  | 0.020 |  |  |  |  |  |  |  |
| >K_H137_I1 |  |  |  |  |  |  |  | 0.020 |  |  |  |  |  |  |  |
| >K_H138_I1 |  |  |  |  |  |  |  | 0.020 |  |  |  |  |  |  |  |
| >K_H139_I1 |  |  |  |  |  |  |  | 0.039 |  |  |  |  |  |  |  |
| >K_H140_I1 |  |  |  |  |  |  |  | 0.020 |  |  |  |  |  |  |  |
| >K_H141_I1 |  |  |  |  |  |  |  | 0.020 |  |  |  |  |  |  |  |
| >K_H142_I1 |  |  |  |  |  |  |  | 0.020 |  |  |  |  |  |  |  |
| >O_H143_I2 |  |  |  |  |  |  |  |  | 0.020 |  |  |  |  |  |  |
| >O_H144_I2 |  |  |  |  |  |  |  |  | 0.020 |  |  |  |  |  |  |
| >O_H145_I2 |  |  |  |  |  |  |  |  | 0.039 |  |  |  |  |  |  |
| >O_H146_I2 |  |  |  |  |  |  |  |  | 0.020 |  |  |  |  |  |  |
| >O_H147_I2 |  |  |  |  |  |  |  |  | 0.039 |  |  |  |  |  |  |
| >O_H148_I2 |  |  |  |  |  |  |  |  | 0.039 |  |  |  |  |  |  |
| >OP_H149_I2 |  |  |  |  |  |  |  |  | 0.020 | 0.032 |  |  |  |  |  |
| >O_H150_I2 |  |  |  |  |  |  |  |  | 0.098 |  |  |  |  |  |  |
| >O_H151_I1 |  |  |  |  |  |  |  |  | 0.078 |  |  |  |  |  |  |
| >O_H152_I1 |  |  |  |  |  |  |  |  | 0.039 |  |  |  |  |  |  |
| >O_H153_I1 |  |  |  |  |  |  |  |  | 0.020 |  |  |  |  |  |  |
| >P_H154_I2 |  |  |  |  |  |  |  |  |  | 0.032 |  |  |  |  |  |
| >P_H155_I2 |  |  |  |  |  |  |  |  |  | 0.032 |  |  |  |  |  |
| >P_H156_I2 |  |  |  |  |  |  |  |  |  | 0.032 |  |  |  |  |  |
| >P_H157_I1 |  |  |  |  |  |  |  |  |  | 0.032 |  |  |  |  |  |
| >P_H158_I2 |  |  |  |  |  |  |  |  |  | 0.032 |  |  |  |  |  |
| >P_H159_I2 |  |  |  |  |  |  |  |  |  | 0.065 |  |  |  |  |  |
| >P_H160_I1 |  |  |  |  |  |  |  |  |  | 0.097 |  |  |  |  |  |
| >P_H161_?? |  |  |  |  |  |  |  |  |  | 0.032 |  |  |  |  |  |
| >P_H162_I1 |  |  |  |  |  |  |  |  |  | 0.032 |  |  |  |  |  |
| >P_H163_I1 |  |  |  |  |  |  |  |  |  | 0.032 |  |  |  |  |  |
| >P_H164_I1 |  |  |  |  |  |  |  |  |  | 0.097 |  |  |  |  |  |
| >P_H165_I1 |  |  |  |  |  |  |  |  |  | 0.032 |  |  |  |  |  |
| >P_H166_I1 |  |  |  |  |  |  |  |  |  | 0.032 |  |  |  |  |  |
| >N_H167_I1 |  |  |  |  |  |  |  |  |  |  | 0.125 |  |  |  |  |
| >N_H168_I1 |  |  |  |  |  |  |  |  |  |  | 0.063 |  |  |  |  |
| >N_H169_I1 |  |  |  |  |  |  |  |  |  |  | 0.063 |  |  |  |  |
| >N_H170_I1 |  |  |  |  |  |  |  |  |  |  | 0.063 |  |  |  |  |
| >U_H171_I1 |  |  |  |  |  |  |  |  |  |  |  | 0.030 |  |  |  |
| >U_H172_I1 |  |  |  |  |  |  |  |  |  |  |  | 0.030 |  |  |  |
| >U_H173_I2 |  |  |  |  |  |  |  |  |  |  |  | 0.091 |  |  |  |
| >U_H174_I2 |  |  |  |  |  |  |  |  |  |  |  | 0.061 |  |  |  |
| >U_H175_I2 |  |  |  |  |  |  |  |  |  |  |  | 0.030 |  |  |  |
| >U_H176_I2 |  |  |  |  |  |  |  |  |  |  |  | 0.030 |  |  |  |
| >U_H177_I2 |  |  |  |  |  |  |  |  |  |  |  | 0.030 |  |  |  |
| >V_H178_I2 |  |  |  |  |  |  |  |  |  |  |  |  | 0.192 |  |  |
| >V_H179_I2 |  |  |  |  |  |  |  |  |  |  |  |  | 0.192 |  |  |
| >V_H180_I2 |  |  |  |  |  |  |  |  |  |  |  |  | 0.039 |  |  |
| >V_H181_I1 |  |  |  |  |  |  |  |  |  |  |  |  | 0.192 |  |  |
| >V_H182_I1 |  |  |  |  |  |  |  |  |  |  |  |  | 0.077 |  |  |
| >V_H183_I1 |  |  |  |  |  |  |  |  |  |  |  |  | 0.039 |  |  |
| >H_H184_T3 |  |  |  |  |  |  |  |  |  |  |  |  |  | 0.067 |  |
| >H_H185_T3 |  |  |  |  |  |  |  |  |  |  |  |  |  | 0.067 |  |
| >H_H186_T3 |  |  |  |  |  |  |  |  |  |  |  |  |  | 0.067 |  |
| >H_H187_I1 |  |  |  |  |  |  |  |  |  |  |  |  |  | 0.067 |  |
| >H_H188_I2 |  |  |  |  |  |  |  |  |  |  |  |  |  | 0.067 |  |
| >H_H189_I1 |  |  |  |  |  |  |  |  |  |  |  |  |  | 0.067 |  |
| >J_H190_T3 |  |  |  |  |  |  |  |  |  |  |  |  |  |  | 0.030 |
| >J_H191_T3 |  |  |  |  |  |  |  |  |  |  |  |  |  |  | 0.030 |
| >J_H192_T3 |  |  |  |  |  |  |  |  |  |  |  |  |  |  | 0.030 |
| >J_H193_T3 |  |  |  |  |  |  |  |  |  |  |  |  |  |  | 0.061 |
| >J_H194_T3 |  |  |  |  |  |  |  |  |  |  |  |  |  |  | 0.061 |
| >J_H195_T3 |  |  |  |  |  |  |  |  |  |  |  |  |  |  | 0.030 |
| >J_H196_T3 |  |  |  |  |  |  |  |  |  |  |  |  |  |  | 0.061 |
| >J_H197_T3 |  |  |  |  |  |  |  |  |  |  |  |  |  |  | 0.030 |
| >J_H198_T3 |  |  |  |  |  |  |  |  |  |  |  |  |  |  | 0.030 |
| >J_H199_T3 |  |  |  |  |  |  |  |  |  |  |  |  |  |  | 0.242 |
| >J_H200_T3 |  |  |  |  |  |  |  |  |  |  |  |  |  |  | 0.030 |
| >J_H201_T3 |  |  |  |  |  |  |  |  |  |  |  |  |  |  | 0.030 |
| >J_H202_T3 |  |  |  |  |  |  |  |  |  |  |  |  |  |  | 0.030 |
| >J_H203_T3 |  |  |  |  |  |  |  |  |  |  |  |  |  |  | 0.030 |
| >J_H204_T3 |  |  |  |  |  |  |  |  |  |  |  |  |  |  | 0.061 |
| >J_H205_T3 |  |  |  |  |  |  |  |  |  |  |  |  |  |  | 0.030 |
| >J_H206_T3 |  |  |  |  |  |  |  |  |  |  |  |  |  |  | 0.030 |
| >J_H207_T3 |  |  |  |  |  |  |  |  |  |  |  |  |  |  | 0.030 |
