# Supplementary material for: Legacy of draught cattle breeds of South India: Insights into population structure, genetic admixture and maternal origin
Source: PLoS One. 2021 May 24;16(5):e0246497. doi: 10.1371/journal.pone.0246497 (PMC8143428; doi:10.1371/journal.pone.0246497)
Supplement: S1 File — (DOCX) [file pone.0246497.s009.docx]

**S1 File.** **Brief description of characteristics of South Indian cattle breeds**

***Bargur,*** the hill cattle of Tamil Nadu are distributed in and around the Bargur hills, Bhavani Taluk of Erode district of Tamil Nadu. Animals of this breed are also scarcely distributed in Koluthar Taluk of Salem district in Tamil Nadu. They are smaller and more compact, with not so prominent forehead as observed in other Mysore type breeds. They have distinct morphological appearance due to their coat colour, typical of being red and white or red with white spots. These cattle are known for their speed and endurance in trotting, but are very fiery, restive and difficult to train [42,53,54].

***Kangayam*** cattle is an excellent draught cattle breed and are distributed in Kangayam, Dharapurm, Perundurai, Karur and Palani areas of Tamil Nadu located in southern India. Kangayam is a hardy breed suitable for all forms of agricultural operations and carting. The bullocks are high power animals with a power of 0.8hp per pair of animals. Kangayam can produce moderate amount of milk with the lactation yield ranging from 342 to 1455 litres [51]. The breed is well adapted to drought prone areas and can thrive under scanty rations [50].

***Umblachery*** is an excellent draught cattle breed reared in the eastern coastal areas of Tamil Nadu state, particularly in Thiruvarur and parts of Nagapattinam districts [63]. These are medium sized cattle, selectively bred for short stature and suitability to work in marshy paddy fields.

***Pulikulam*** cattle are maintained as migratory herds in Madurai, Sivagangai and Virudhunagar districts of Tamil Nadu state. They have predominantly white or greyish coat colour and are mostly used for agricultural operations and rural transport. These cattle are well-known for their utilities as a sport animal for Jallikattu (a traditional bull embracing sport) and penning (a practice of manuring by keeping the cattle overnight in open agricultural fields) [45].

***Alambadi*** cattle are bred in the hilly tracts of Salem and Erode districts of Tamil Nadu with the animals grazed mostly in the forest areas. These animals are considered as offshoot of the Hallikar cattle and are hardy and very active. They are good draught animals and are known for carting and mhote work. The cows are poor milkers [42].

***Hallikar*** breed of cattle is distributed in the districts of Mysore, Tumkur, Chitradurga, Mandya, Kolar and Hassan in Karnataka state of South India. Hallikar is considered as the progenitor of most Mysore type breeds. The animals are generally sturdy, powerful and docile capable of working for an average duration of 8.18h/day. The average load pulling capacity of Hallikar bullocks was 2.75 tonnes with an average speed of 5kmph [55].

***Deoni*** cattle is distributed in Bidar and Gulbarga districts of Karnataka; Medak district of Andhra Pradesh; Latur, Parbhani, Nanded and Osmanabad districts of Maharashtra. There are 3 strains of Deoni based on coat colour variations: Wannera (white coat colour with black colour at the sides of the face), Shevera (white body with irregular black spots) and Balankya (white coat colour with black spots on the lower side of the body). Deoni is a dual-purpose breed, capable of working for 7-8 hours a day and producing moderate amounts of milk [52,64].

***Ongole*** cattle is distributed in the Nellore and Guntur districts of Andhra Pradesh. These are large-sized animals and are known for both work and milk production. The bullocks are very powerful and are suitable for ploughing and heavy cart work, but are not considered favourably for speed and trotting purposes. Ongole cattle are average milk producers [9].

***Vechur and Punganur*** are unique to South India and are probably among the smallest cattle breeds of the world, with the average height of adults ranging from 60-100 cm and body weight of 125-200 kg [65,66]. Vechur cattle are distributed in and around Vaikom in the Kottayam District of Kerala. Punganur cattle are distributed in Vayalpad, Madanaspalli and

Palamaneru taluks of Chittoor district of Andhra Pradesh. Vechur and Punganur cattle breeds are distinct from rest of the South Indian breeds, not only in terms of morphology but also in terms of milk production efficiency. The cows are considered to be efficient milk producers relative to their size, with peak milk yield scaling up to 3-4 kg/day [65,66].

**Additional Supplementary Material References**

63. Rajendran R, Raja TV, Thiruvenkadan AK, Nainar MA, Thangaraju P. Morphobiometrical characteristics and management of Umblachery cattle from coastal region of Tamilnadu, India. Livestock Research for Rural Development. 2008; 20: 40.

64. Kuralkar SV, Bankar PS, Chopade MM, Kuralkar P, Dhaware SA. Phenotypic characteristics, production and reproduction performance of Deoni cattle in its native tract. Indian Journal of Animal Sciences, 2014; 84 (1): 75–77.

65. Nath NM. Punganur – The Miniature *Bos indicus* cattle. Animal Genetic Resources Information. 1993; 11: 54-59.

66. Iype S. The Vechur Cattle of Kerala. Animal Genetic Resources Information. 1996; 18: 59-63.
