# Supplementary material for: Legacy of draught cattle breeds of South India: Insights into population structure, genetic admixture and maternal origin
Source: PLoS One. 2021 May 24;16(5):e0246497. doi: 10.1371/journal.pone.0246497 (PMC8143428; doi:10.1371/journal.pone.0246497)
Supplement: S2 File — (DOCX) [file pone.0246497.s010.docx]

**S2 File. Representative pictures of investigated Zebu cattle breeds of South India**

**Bargur**

**
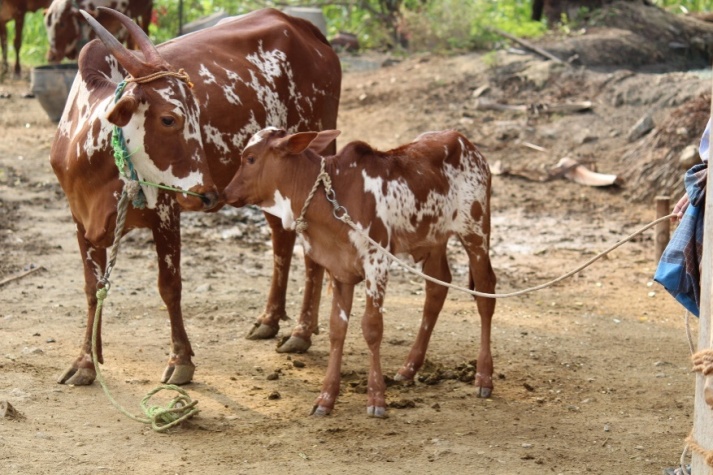
**

**
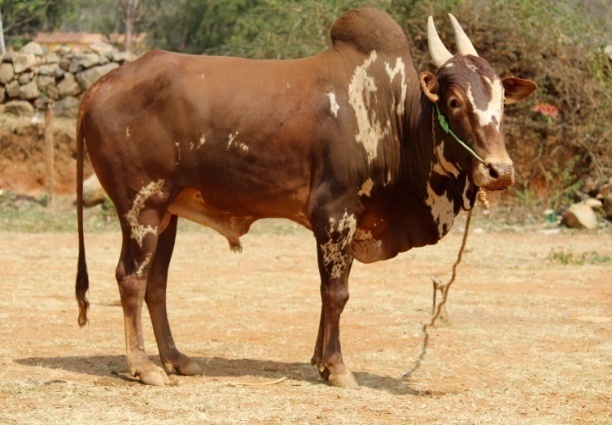
**

**Kangayam**

**
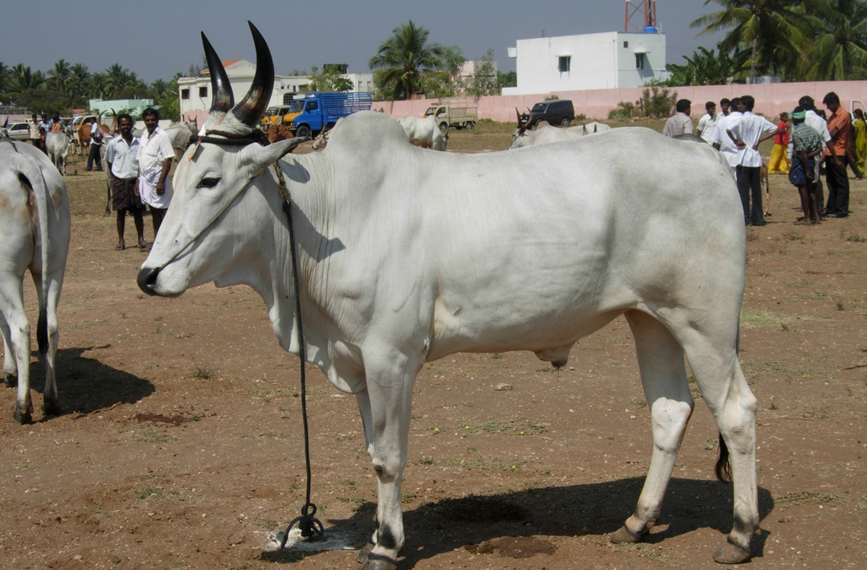
**


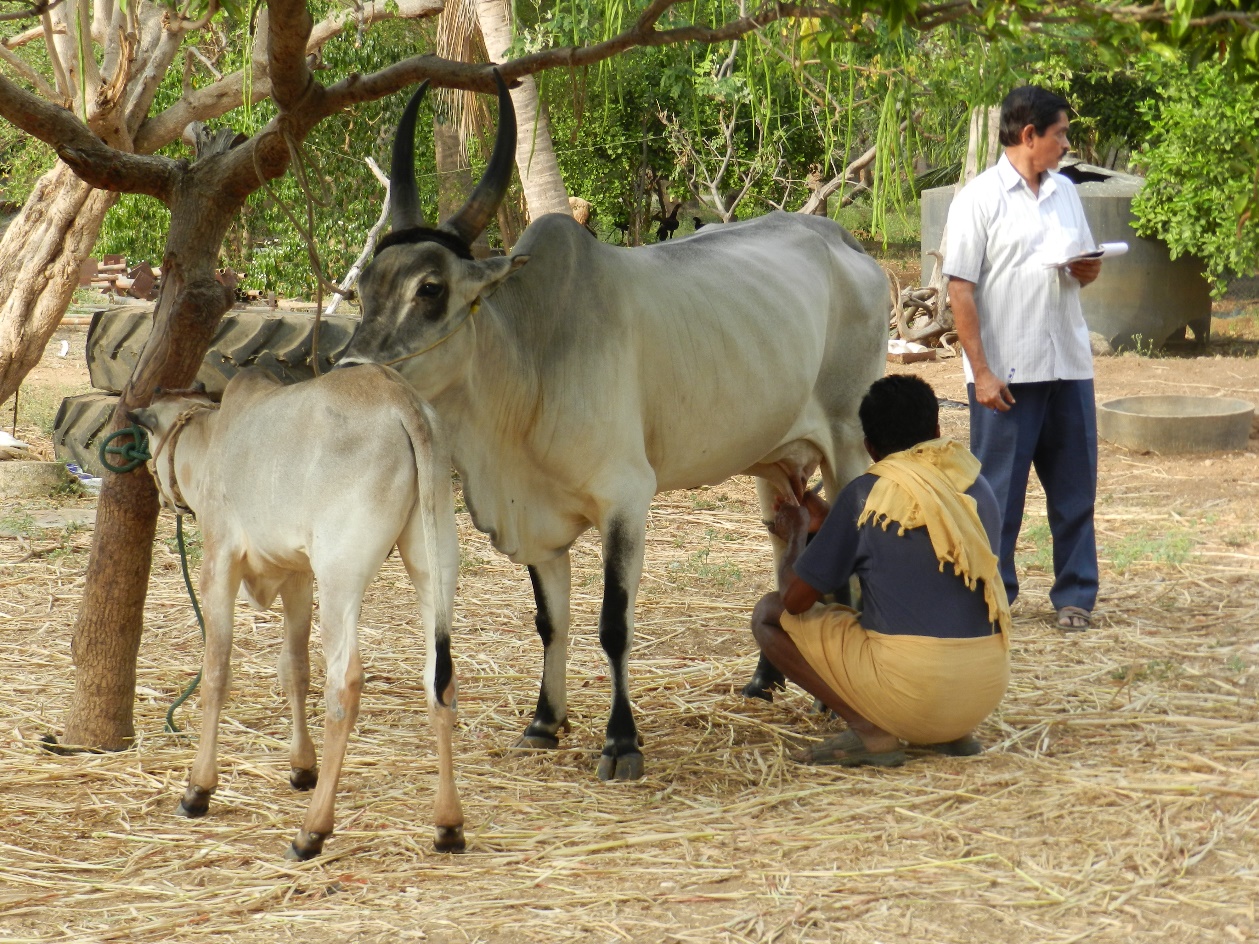


**Pulikulam**


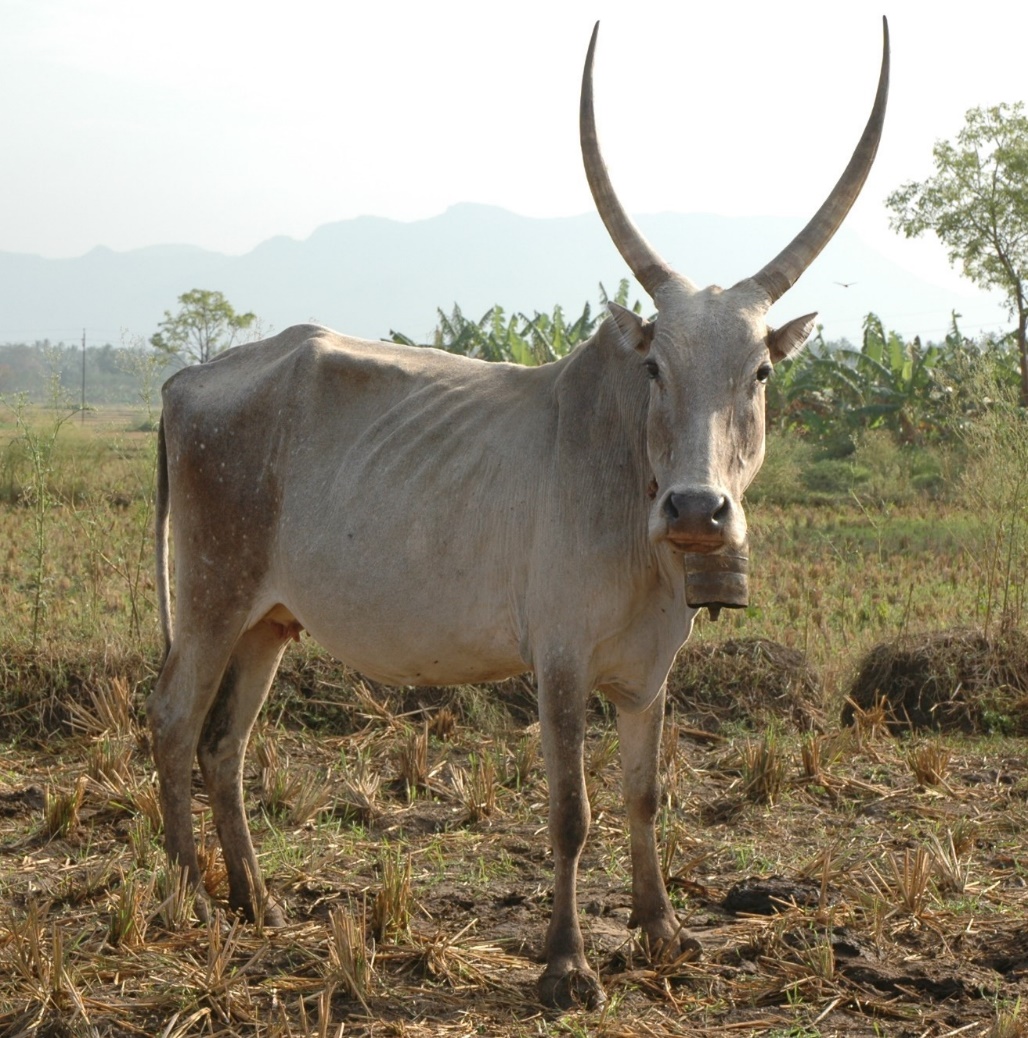


**
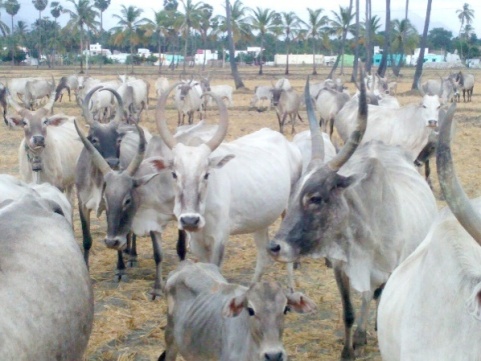
**

**Umblachery**


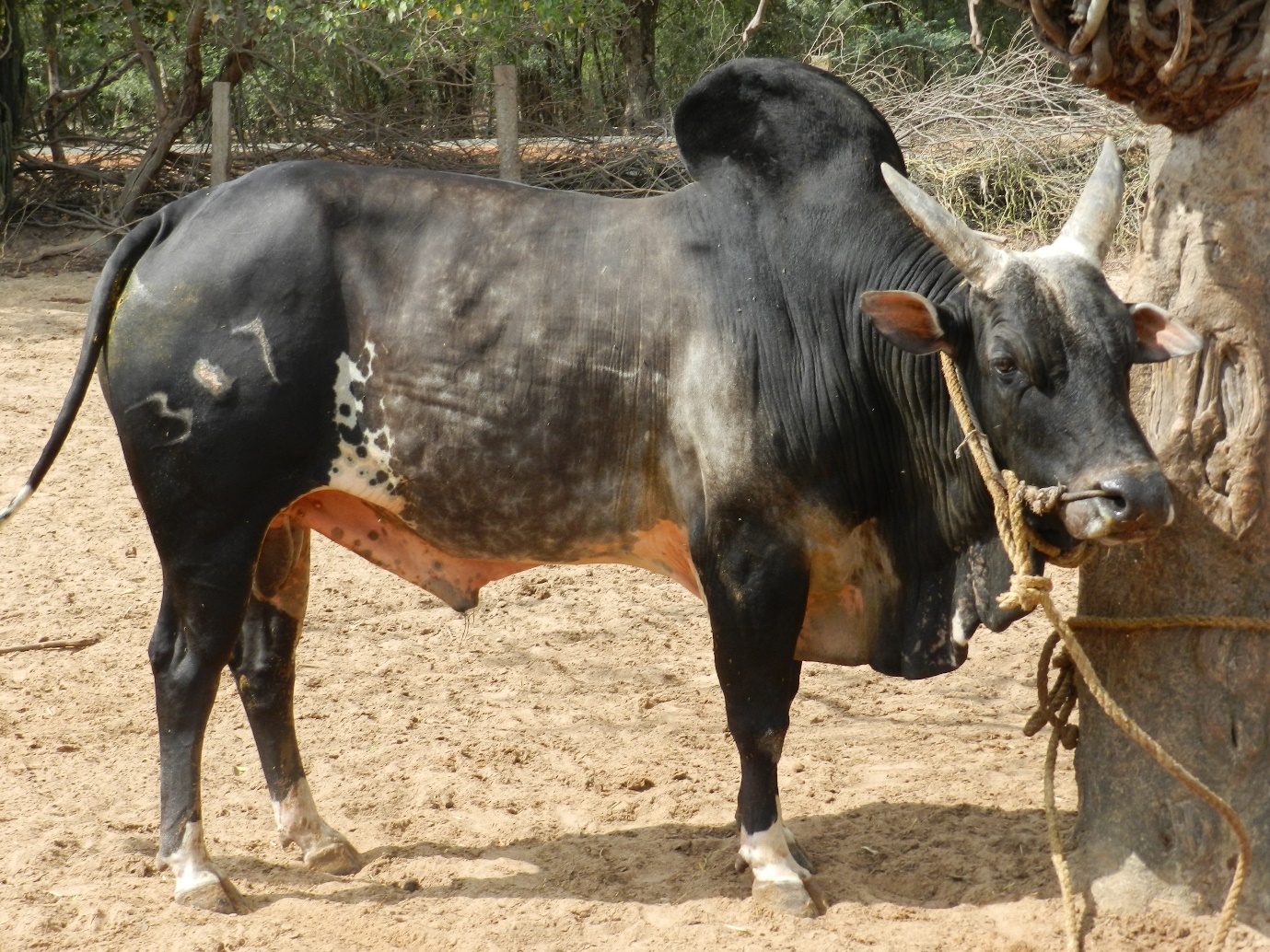


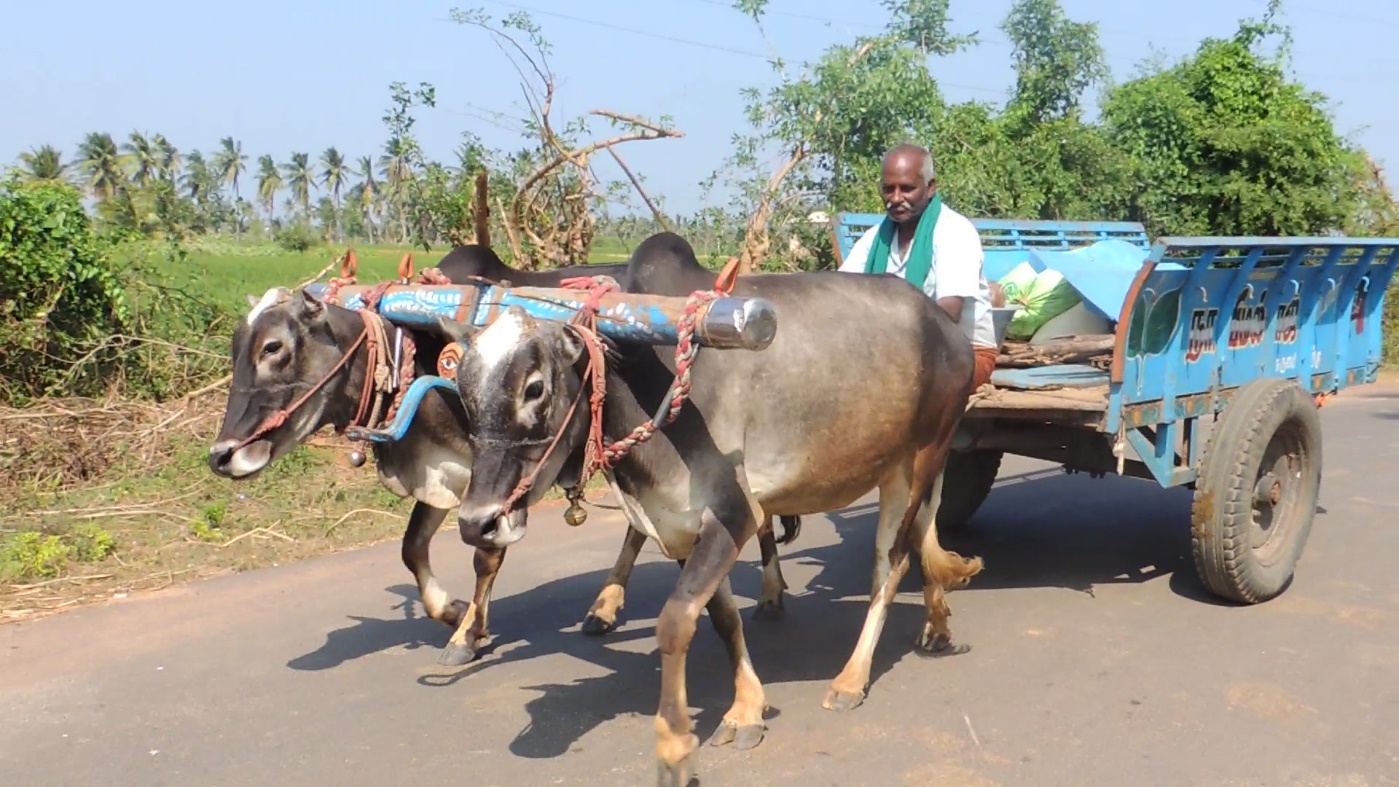


**Alambadi**


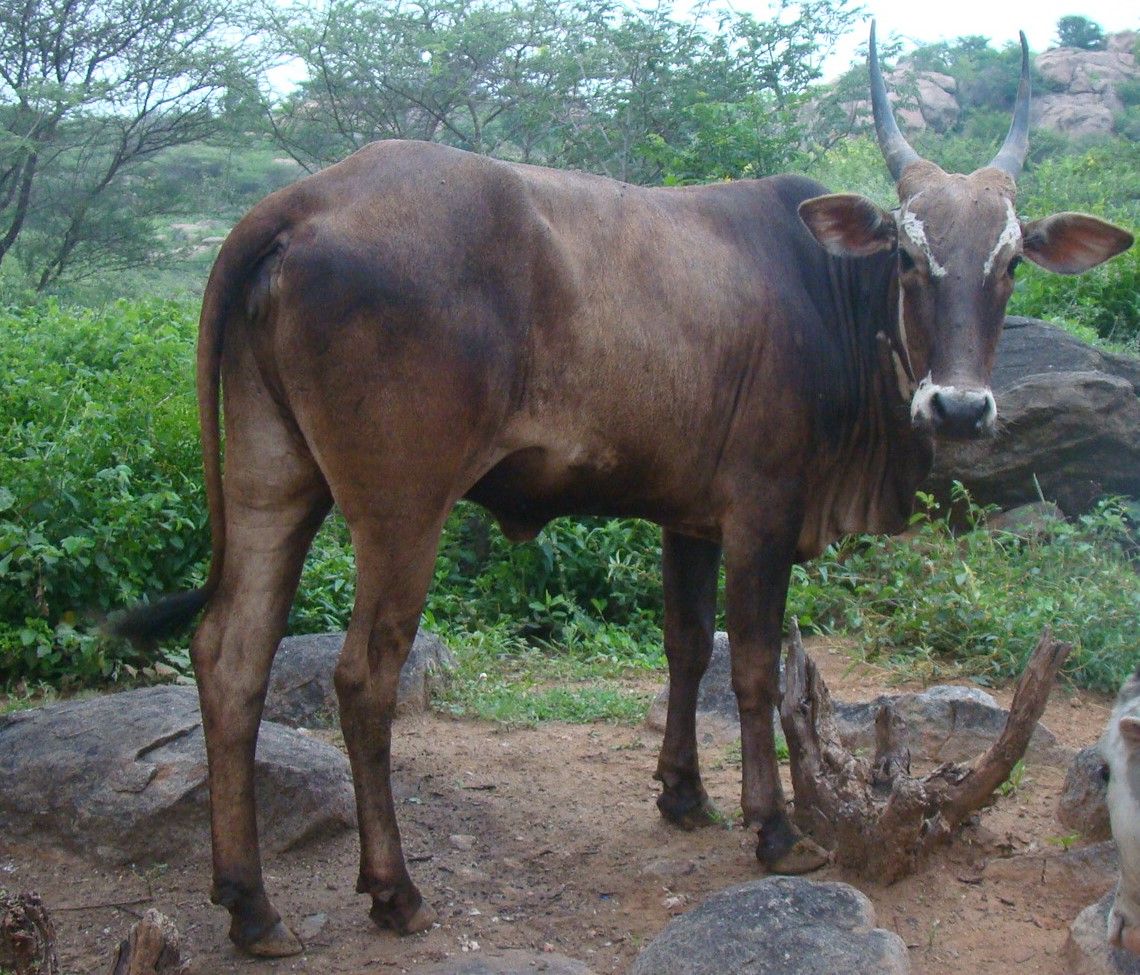


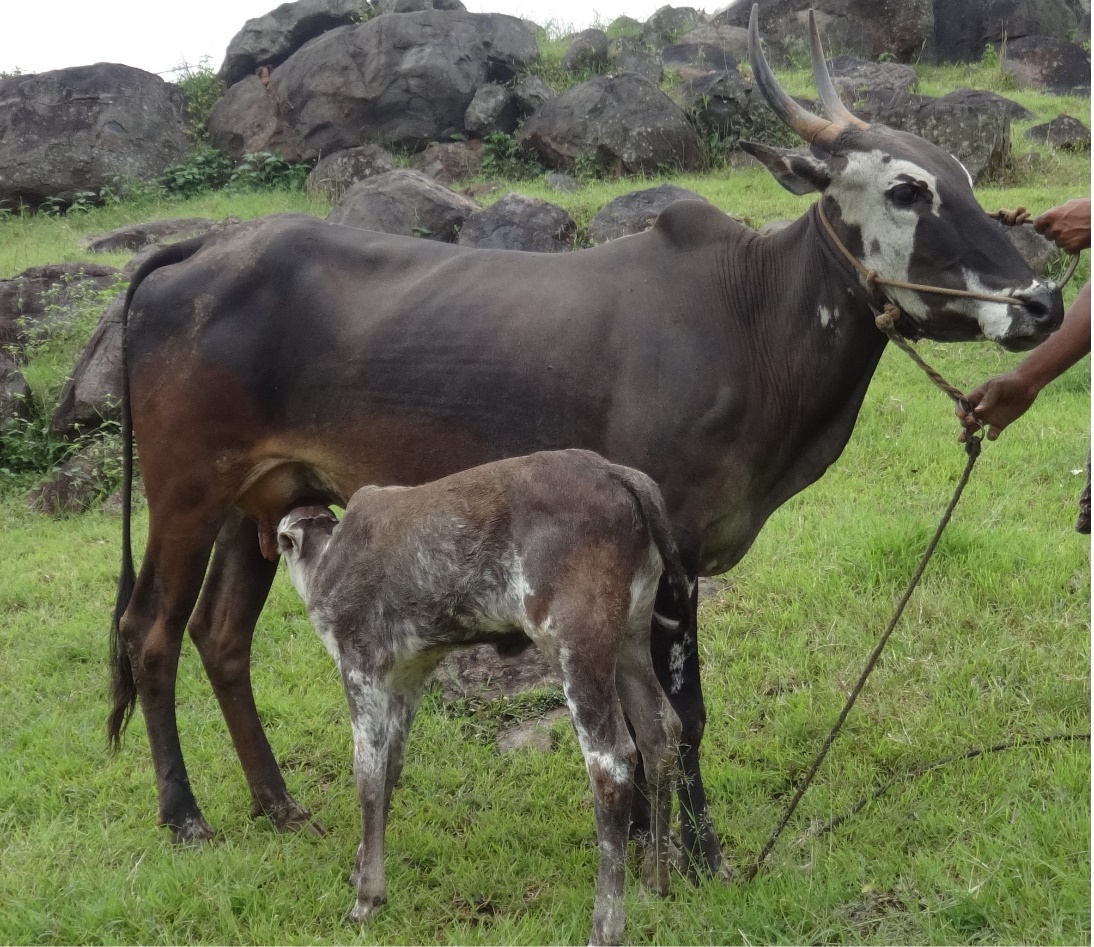


**Hallikar**


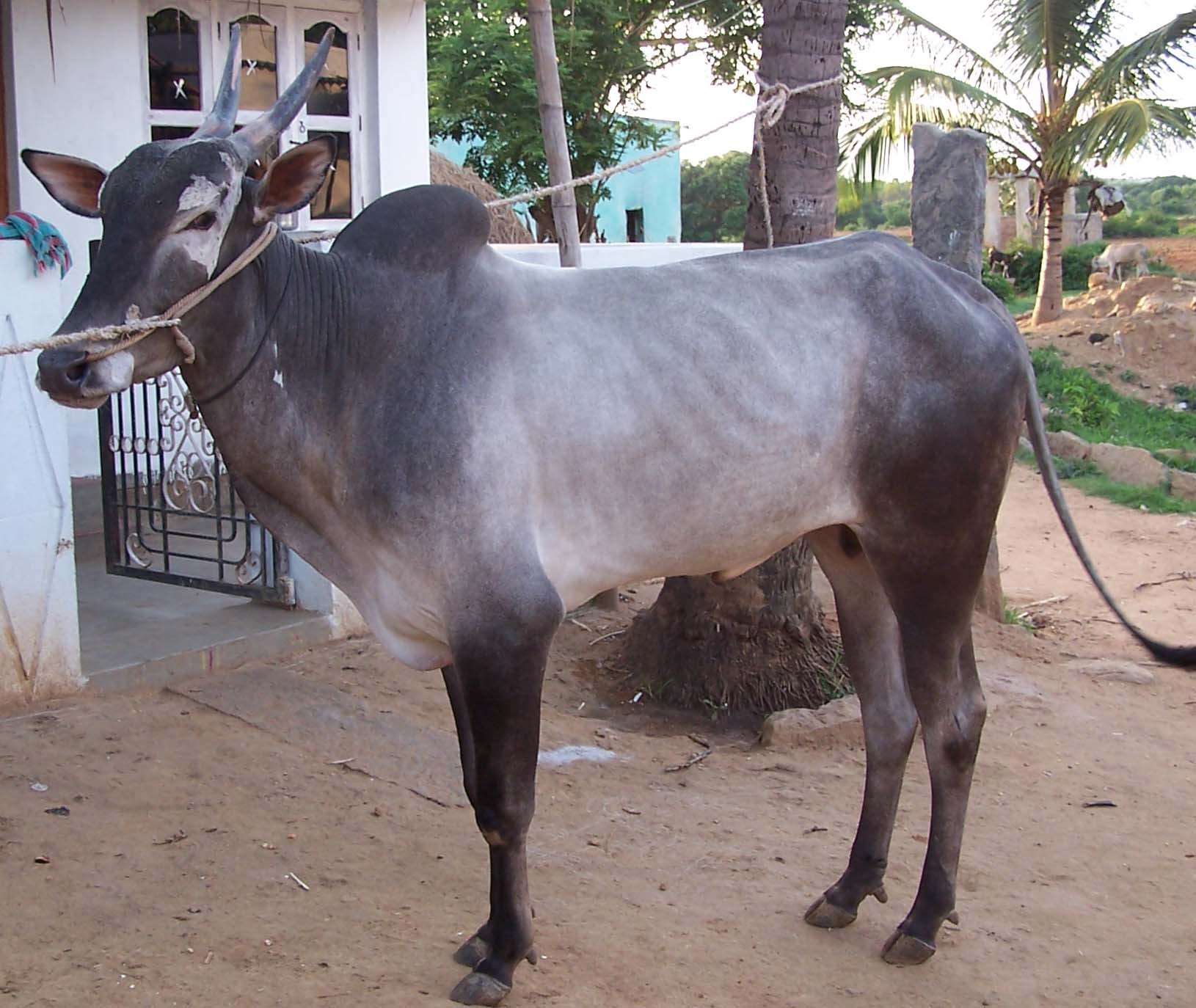


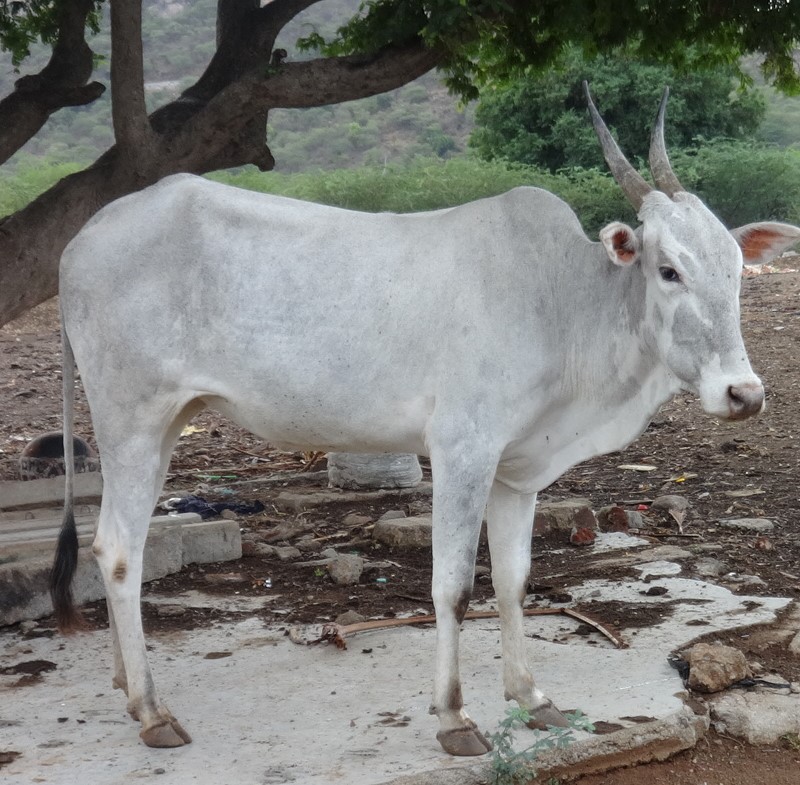


**Deoni**


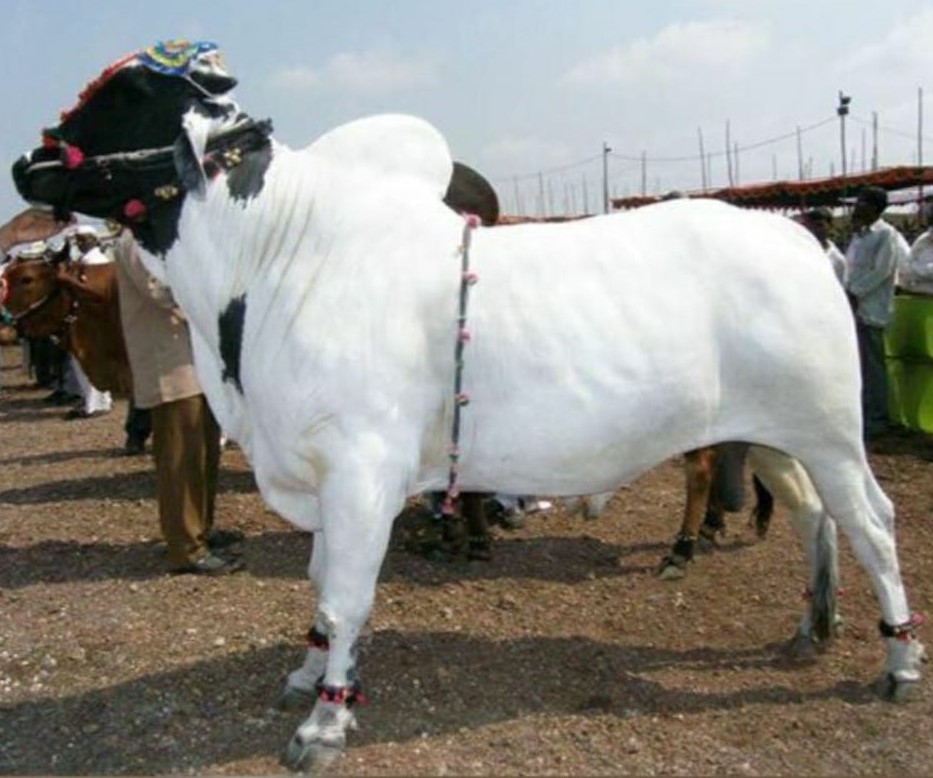


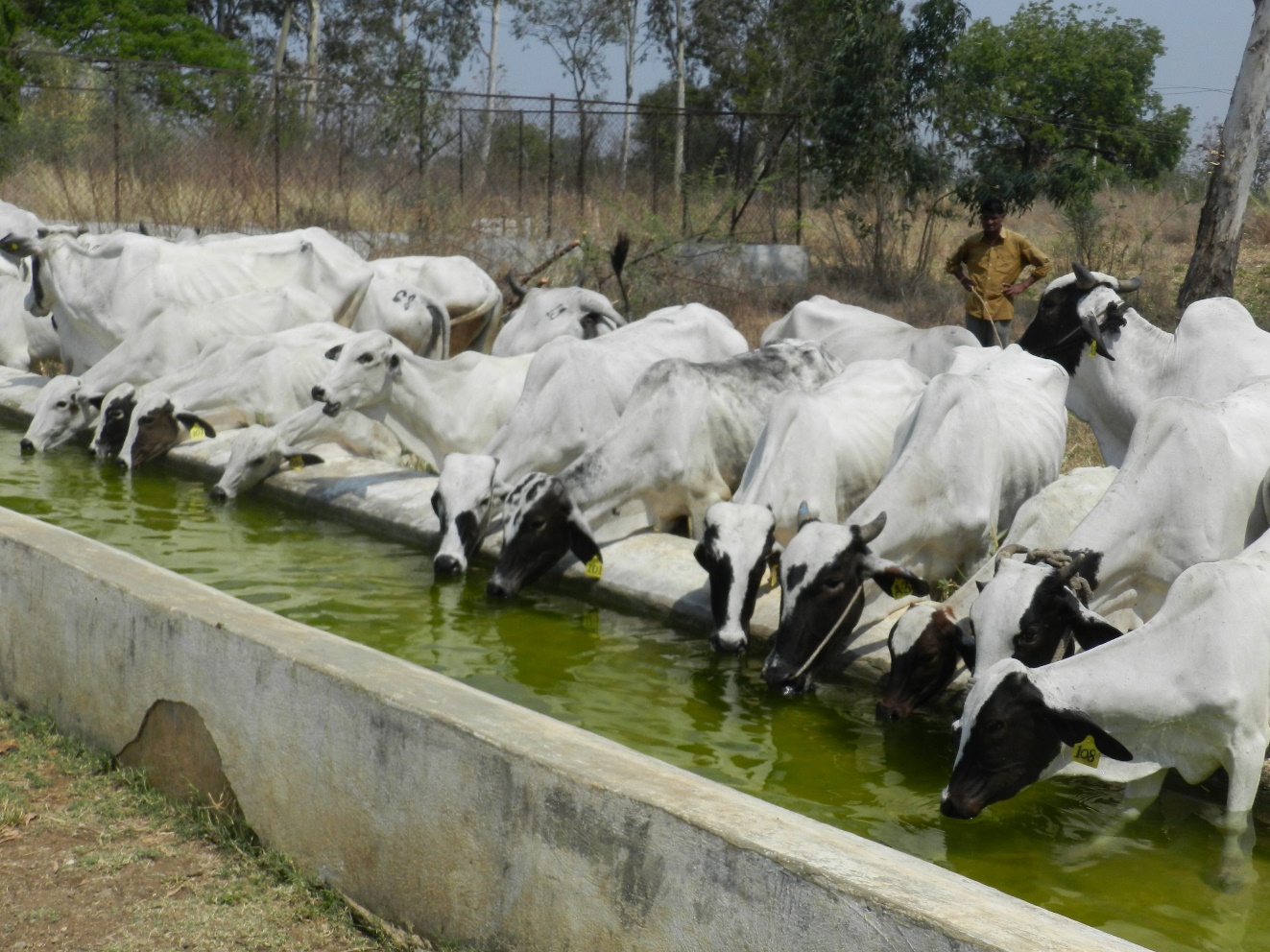


**Ongole**


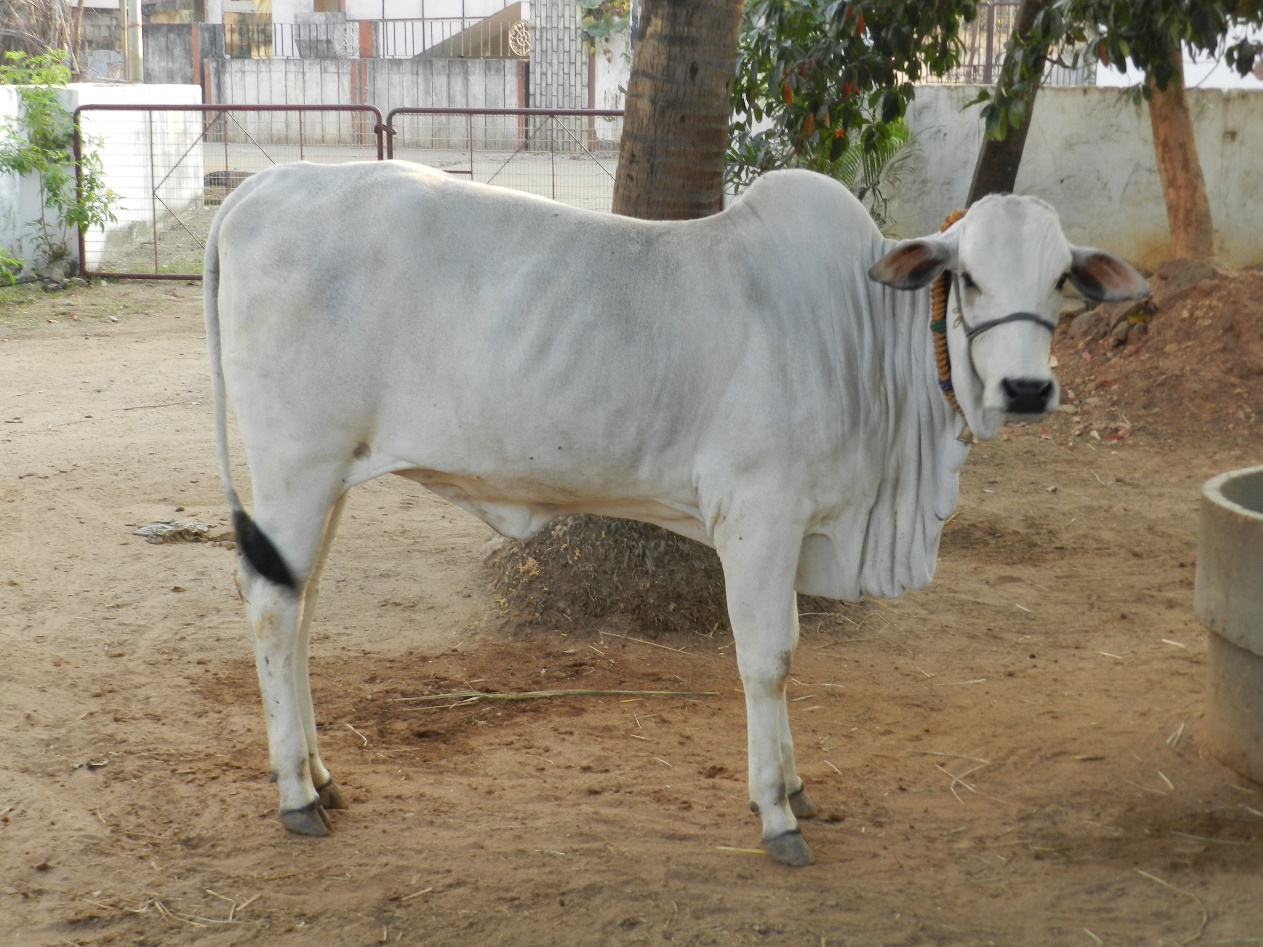


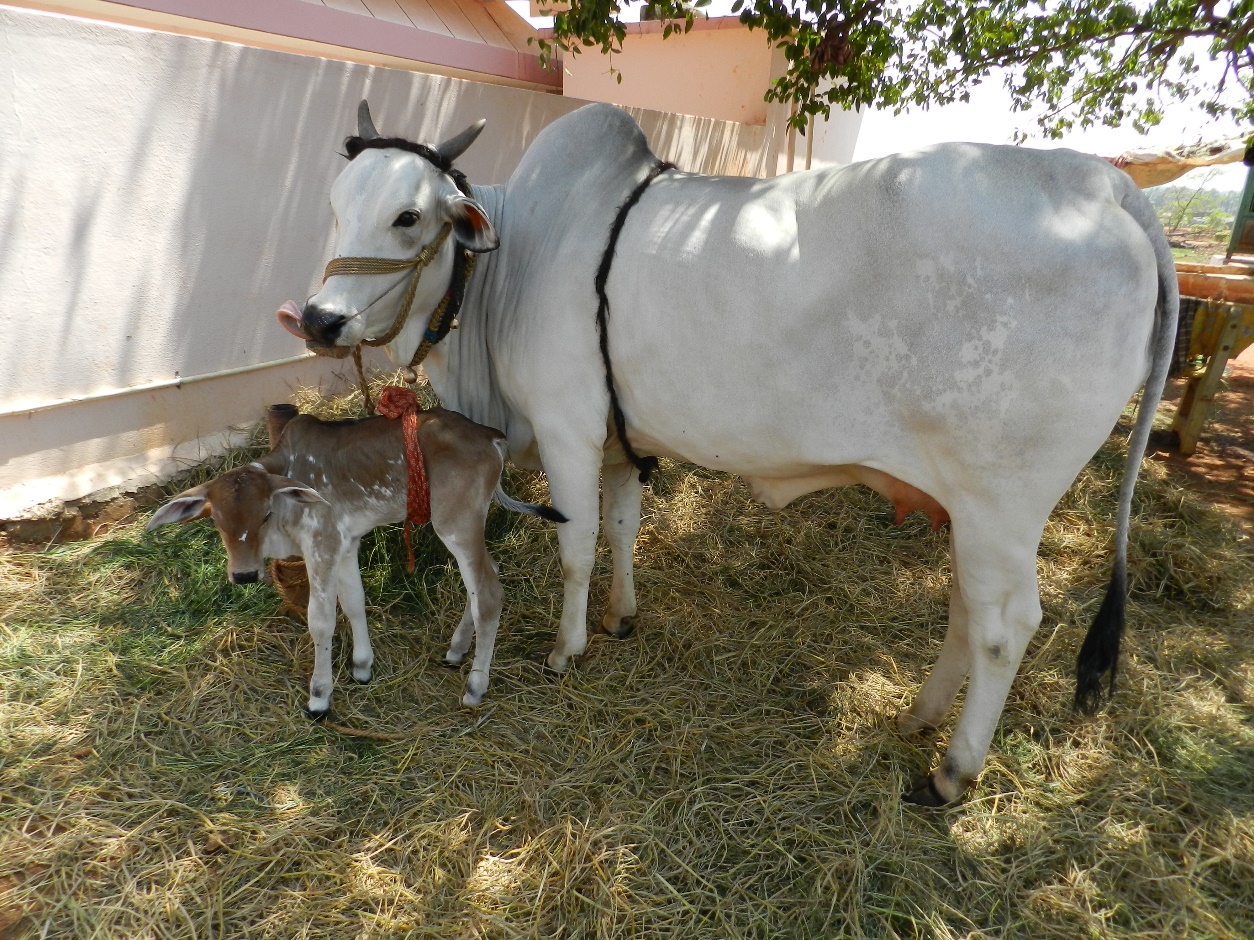


**Punganur**


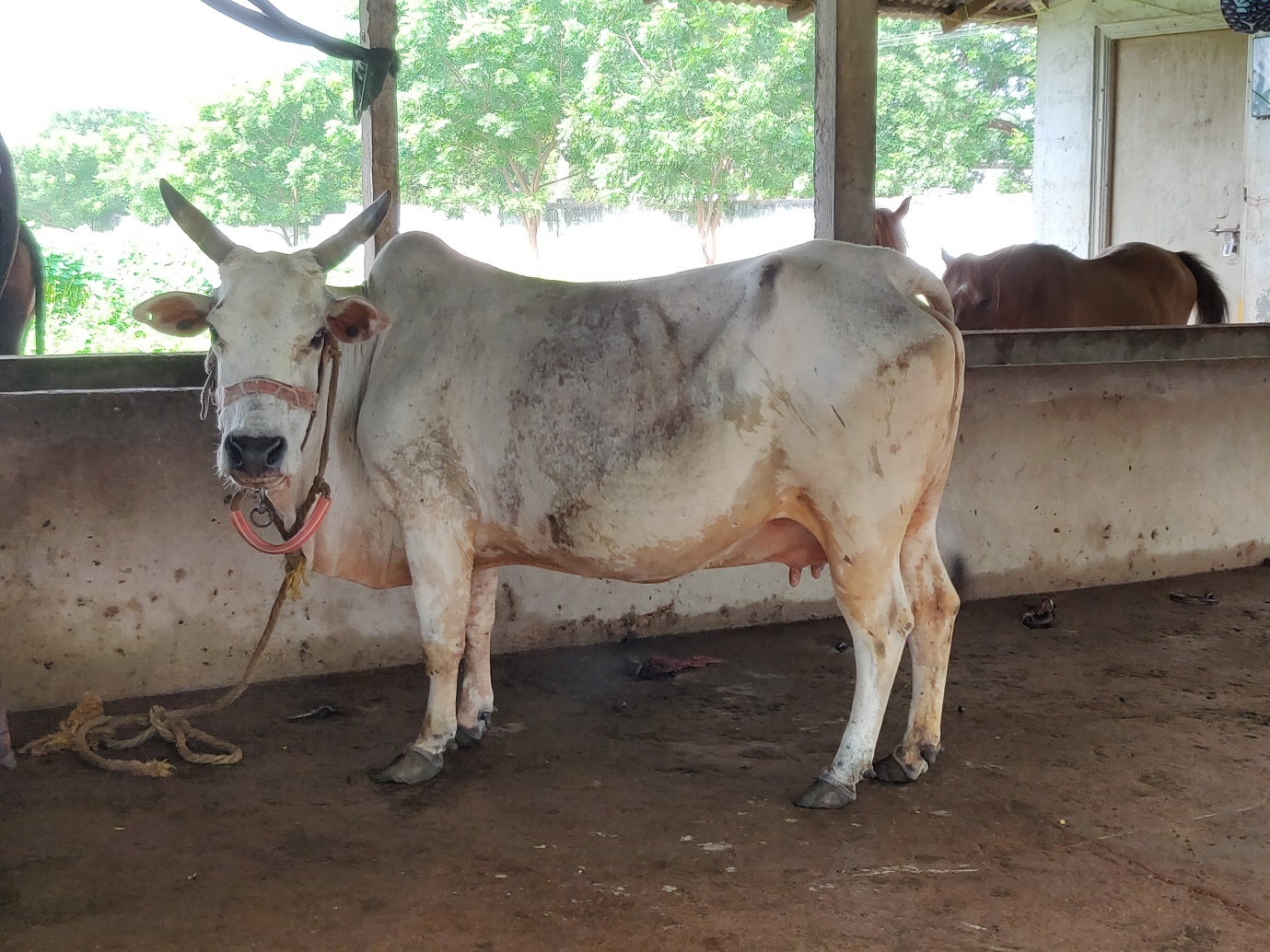


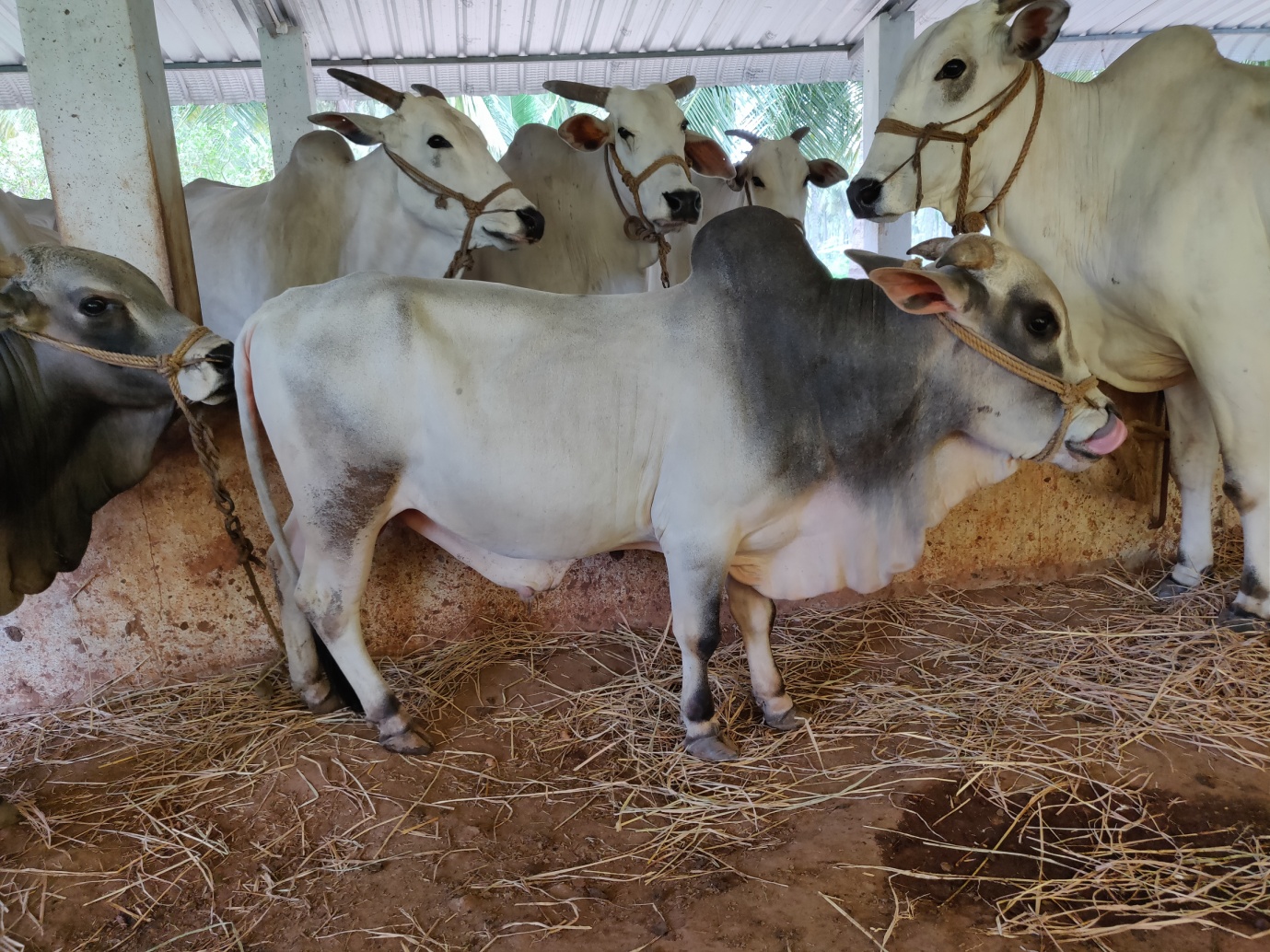


**Vechur**


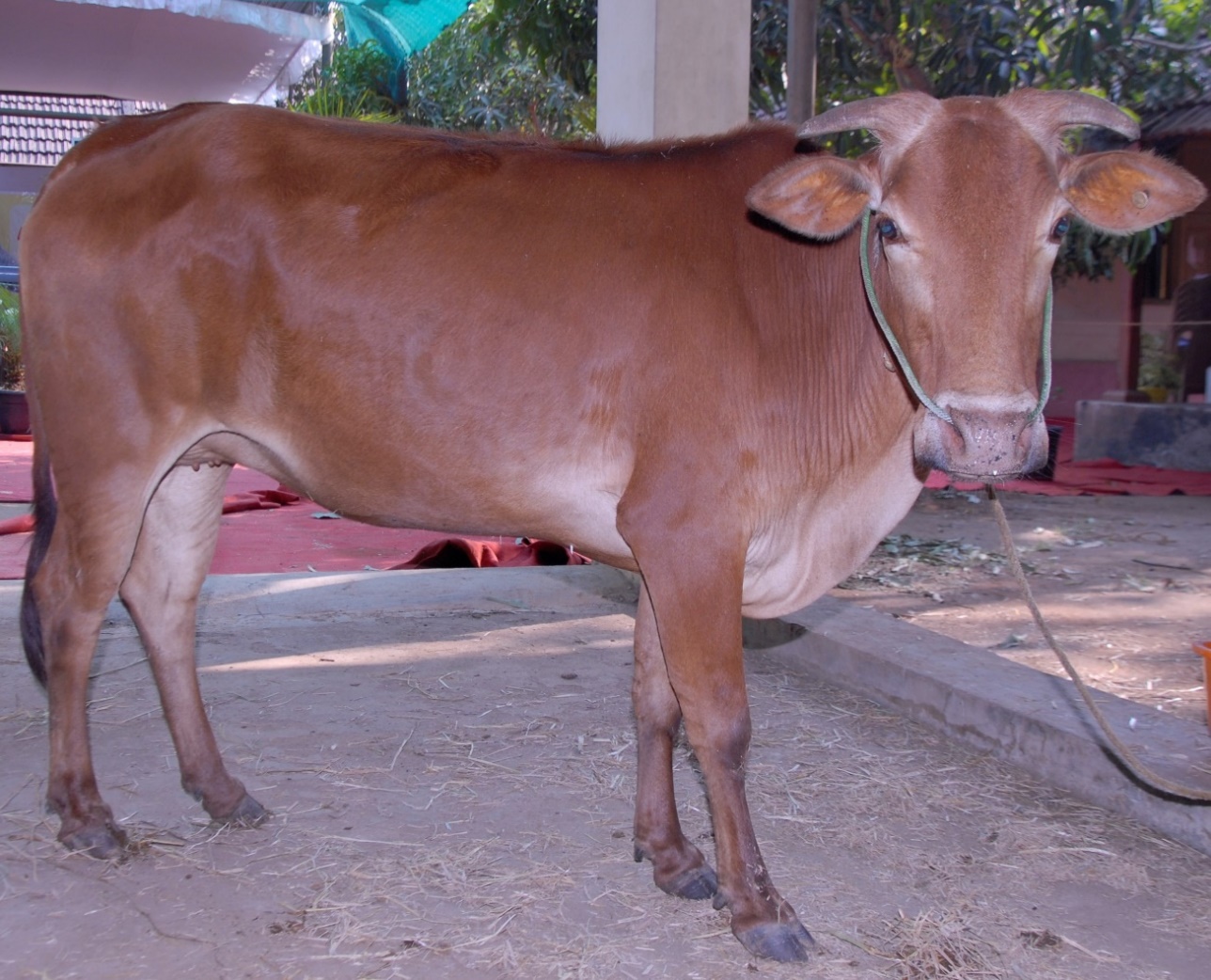


**
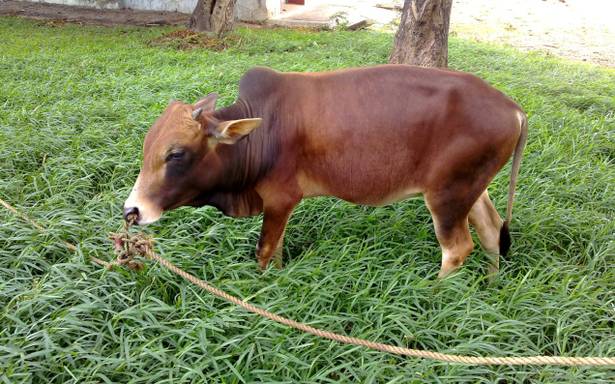
**
